# MicroRNA in combination with HER2-targeting drugs reduces breast cancer cell viability in vitro

**DOI:** 10.1038/s41598-021-90385-2

**Published:** 2021-05-25

**Authors:** Lisa Svartdal Normann, Miriam Ragle Aure, Suvi-Katri Leivonen, Mads Haugland Haugen, Vesa Hongisto, Vessela N. Kristensen, Gunhild Mari Mælandsmo, Kristine Kleivi Sahlberg

**Affiliations:** 1 Department of Research and Innovation, Vestre Viken Hospital Trust, P.O. Box 800, 3004 Drammen, Norway; 2 Department of Tumor Biology, Institute for Cancer Research, The Norwegian Radium Hospital, Oslo University Hospital, Oslo, Norway; 3 Department of Medical Genetics, Institute of Clinical Medicine, Faculty of Medicine, University of Oslo, Oslo, Norway; 4 Applied Tumor Genomics Research Program, Medical Faculty, University of Helsinki, Helsinki, Finland; 5 Division of Toxicology, Misvik Biology, Turku, Finland; 6 Division of Medicine, Department of Clinical Molecular Biology (EpiGen), Akershus University Hospital, Lørenskog, Norway; 7 Institute for Medical Biology, Faculty of Health Sciences, UiT—The Arctic University of Norway, Tromsø, Norway

**Keywords:** Breast cancer, High-throughput screening, Breast cancer, miRNAs

## Abstract

HER2-positive (HER2 +) breast cancer patients that do not respond to targeted treatment have a poor prognosis. The effects of targeted treatment on endogenous microRNA (miRNA) expression levels are unclear. We report that responsive HER2 + breast cancer cell lines had a higher number of miRNAs with altered expression after treatment with trastuzumab and lapatinib compared to poorly responsive cell lines. To evaluate whether miRNAs can sensitize HER2 + cells to treatment, we performed a high-throughput screen of 1626 miRNA mimics and inhibitors in combination with trastuzumab and lapatinib in HER2 + breast cancer cells. We identified eight miRNA mimics sensitizing cells to targeted treatment, *miR-101-5p*, *mir-518a-5p*, *miR-19b-2-5p*, *miR-1237-3p*, *miR-29a-3p*, *miR-29c-3p*, *miR-106a-5p*, and *miR-744-3p*. A higher expression of *miR-101-5p* predicted better prognosis in patients with HER2 + breast cancer (OS: *p* = 0.039; BCSS: *p* = 0.012), supporting the tumor-suppressing role of this miRNA. In conclusion, we have identified miRNAs that sensitize HER2 + breast cancer cells to targeted therapy. This indicates the potential of combining targeted drugs with miRNAs to improve current treatments for HER2 + breast cancers.

## Introduction

Breast cancer patients with overexpression of the human epidermal growth factor receptor 2 (HER2) are classified as HER2-positive (HER2 +). This subtype of breast cancer shows an aggressive path with intracellular signaling resulting in increased cell survival and proliferation. The receptor is encoded by the *ERBB2* gene, which is amplified in about 20% of all breast cancer patients^1–3^. Over the last two decades several HER2-targeting drugs have been introduced. The most pronounced drug is the monoclonal antibody trastuzumab, which has improved disease free survival and outcome for HER2 + patients when used adjuvantly in combination with chemotherapy^1,4–6^. Despite the introduction of trastuzumab, many patients respond poorly to the treatment or develop resistance, and experience disease progression or relapse. To overcome the lack of response to treatment, other HER2-targeting drugs, such as the small molecule inhibitor lapatinib, the monoclonal antibody pertuzumab and the antibody—cytotoxic agent conjugate trastuzumab emtansine (TDM-1), have been introduced in combination with chemotherapy and other HER2-targeted drugs. Recently, the Food and Drug Administration (FDA) approved the use of the tyrosine kinase inhibitor tucatinib in combination with trastuzumab and capecitabine, and the monoclonal antibody margetuximab-cmkb in combination with chemotherapy as third line treatments based on the effects shown in the HER2CLIMB trial (NCT02614794) and the SOPHIA trial (NCT02492711), respectively. Despite new and improved regimens entering the clinic, there is still a need for continuous exploration of better treatment strategies for patients that respond poorly to the treatment given.

MicroRNAs (miRNAs) are short, single stranded oligonucleotides that regulate gene expression at a posttranscriptional level. These non-coding RNA molecules bind complementary messenger RNA (mRNA) molecules, causing either blockage of translation or degradation of the mRNA. miRNAs may have oncogenic or tumor suppressive functions depending on whether they bind tumor suppressor or oncogenic mRNAs, respectively. Furthermore, one miRNA can target different mRNAs, and several miRNAs can target the same mRNA. In breast cancer, multiple pre-clinical and prognostic implications of miRNAs have been reported. These include the identification of miRNAs essential for HER2-positive breast cancer cell growth^7^, miRNAs that regulate estrogen receptor signaling^8^, miRNAs that increase the proliferation of breast cancer cell lines^9^, miRNAs acting as metastasis suppressors in breast cancer^10^, miRNAs positively regulating cell migration and invasion^11^, and previously we linked a high expression level of miR-29c to disease specific survival^12^.

miRNAs also show a therapeutic potential either alone or in combination with other treatment strategies. Promising effects are reported for miRNAs sensitizing tumors to radiation^13,14^, chemotherapy^15,16^ and targeted treatment^17,18^ in multiple cancer types. A phase I dose escalating trial using TargomiRs (minicells loaded with miR-16 mimics) in malignant pleural mesothelioma and non-small cell lung cancer patients concluded on an acceptable safety profile and early signs of therapeutic activity^19^ (NCT02369198). This latter example lays a promising ground for further work on miRNA therapy.

In the present study, we aim to explore whether miRNAs may sensitize cancer cells to HER2-targeted treatment. We hypothesize that the treatment of HER2 + breast cancer cell lines that respond poorly to trastuzumab and lapatinib can be improved by adding miRNA mimics and inhibitors as therapeutic agents. To identify miRNAs that may sensitize to treatment, we have performed a high-throughput screen of miRNA mimics and inhibitors in HER2 + cell lines in combination with HER2-targeted treatment. Hits from the screen were explored and we here present the most relevant miRNAs to include in combinatorial treatment with trastuzumab and lapatinib.

## Results

### Deregulations of miRNA expression after HER2-targeted treatment

The level of specific miRNAs in a cell is context-dependent and may vary due to cellular processes or in response to other stimuli. Therefore, we wanted to study to what extent treatment with HER2-targeted drugs impact the endogenous miRNA expression levels in breast cancer cells. Four HER2 + breast cancer cell lines were tested, of which two were responsive to trastuzumab and lapatinib (SKBR3 and BT-474) and two were poorly responding (SUM190PT and KPL4)^20^. We identified miRNAs differentially expressed before versus after treatment considering three treatment groups (trastuzumab, lapatinib or the combination of trastuzumab and lapatinib).

Altogether, the expressions of 12 miRNAs were significantly changed when studying the miRNA expression levels in all cell lines combined between treatment groups (Kruskal–Wallis *p*-value < 0.05; Table 1). For the responsive cell lines, 36 miRNAs changed in expression levels due to treatment. Nine of these overlap with the miRNAs regulated when combining results from all four cell lines, indicating that the effects in the responsive lines are substantial enough to withstand the smaller or lack of effects in the poorly responsive cell lines. For the poorly responding cell lines, the expression level of only one miRNA, miR-1268, changed significantly due to treatment (*p* = 0.05; Table 1). The higher number of altered miRNAs in the responsive versus poorly responsive cells may reflect some of the intracellular processes leading to cell death or cell growth inhibition upon treatment with HER2-targeted drugs.

**Table 1:** miRNAs responding to HER2-targeted treatment (miRNAs are ordered alphabetically).

Cell lines	miRNAs
All four cell lines BT-474, SKBR3, KPL4, SUM190PT	hsa-let-7b* (*p* = 0.02)*,* **hsa-miR-1207-5p** (*p* = 0.02)*,* **hsa-miR-1236** (*p* = 0.02)*,* hsa-miR-1307 (*p* = 0.04)*,* **hsa-miR-134** (*p* = 0.03)*,* hsa-miR-15b (*p* = 0.04)*,* hsa-miR-25* (*p* = 5E-3)*,* hsa-miR-2861 (*p* = 0.04)*,* **hsa-miR-3656** (*p* = 0.03)*,* **hsa-miR-3663-3p** (*p* = 0.03)*,* **hsa-miR-3940** (*p* = 0.02)*,* **hsa-miR-885-5p** (*p* = 0.02)
Responding cell lines BT-474, SKBR3	hsa-let-7b* (*p* = 0.02)*,* **hsa-miR-1207-5p***,* hsa-miR-1225-5p (*p* = 0.01)*,* hsa-miR-1226* (*p* = 0.01)*,* **hsa-miR-1236** (*p* = 0.02)*,* **hsa-miR-134** (*p* = 1E-3)*,* hsa-miR-150* (*p* = 0.02)*,* hsa-miR-15b* (*p* = 0.05)*,* hsa-miR-1915 (*p* = 0.03)*,* hsa-miR-25* (*p* = 0.02)*,* hsa-miR-3188 (*p* = 0.02)*,* hsa-miR-320a (*p* = 0.03)*,* hsa-miR-320b (*p* = 0.01)*,* hsa-miR-320c (*p* = 0.02)*,* hsa-miR-320d (*p* = 0.02)*,* hsa-miR-320e (*p* = 0.03)*,* hsa-miR-324-3p (*p* = 0.03)*,* **hsa-miR-3656** (*p* = 0.02)*,* **hsa-miR-3663-3p** (*p* = 0.01)*,* hsa-miR-3665 (*p* = 4E-3)*,* hsa-miR-371-5p (*p* = 3E-3)*,* **hsa-miR-3940** (*p* = 0.02)*,* hsa-miR-4270 (*p* = 0.02)*,* hsa-miR-4271 (*p* = 0.01)*,* hsa-miR-4281 (*p* = 0.01)*,* hsa-miR-431* (*p* = 0.04)*,* hsa-miR-4327 (*p* = 0.01)*,* hsa-miR-484 (*p* = 0.02)*,* hsa-miR-595 (*p* = 0.05)*,* hsa-miR-602 (*p* = 0.04)*,* hsa-miR-762 (*p* = 0.05)*,* **hsa-miR-885-5p** (*p* = 0.02)*,* hsv1-miR-H18 (*p* = 0.04)*,* hsv2-miR-H10 (*p* = 0.05)*,* hsv2-miR-H6 (*p* = 0.01)*,* kshv-miR-K12-3 (*p* = 0.02)
Poorly responding cell lines KPL4, SUM190PT	hsa-miR-1268 (*p* = 0.05)

*p*-values calculated using a Kruskal–Wallis test. Repeating miRNAs are highlighted in bold font.

Overall, the miRNA regulations were mainly positive, i.e. the drug treatment caused upregulation of certain miRNAs (Supplementary Tables S2-S4). The responsive cell lines not only show the highest number of altered miRNAs, but also the largest magnitude of significant changes in miRNA expression (Supplementary Table S3). Boxplots showing significant changes in miRNA expression are presented in Supplementary Fig. S2. These results show that endogenous miRNA expression is affected by drug treatment which again may have downstream cascading effects.

To examine which pathways the miRNAs with altered expression may be relevant for, we identified predicted mRNA targets using the MicroRNA Target Filter in the Ingenuity Pathway Analysis (IPA) software program and further used these mRNAs to assess any enrichment of specific pathways in the web-based online tool Enrichr^21,22^. The analysis was performed three times; considering all four cell lines, responsive cell lines alone and finally poorly responsive cell lines alone (Supplementary Tables S2-S4). For the responsive cell lines, the predicted mRNA targets of the deregulated miRNAs were significantly enriched for 26 pathways, whereof the MAPK signaling pathway was the most significant (adjusted *p*-value = 0.003; Supplementary Table S3). This could indicate that one of the effects of HER2-targeting drugs is alterations in a certain set of miRNAs, which further affects the MAPK signaling pathway. When examining all four cell lines and the poorly responsive cell lines alone, no significant (adjusted *p*-value < 0.05) pathways were identified.

### miRNAs sensitizing HER2 + cells to HER2-targeted treatment

To identify miRNAs that sensitize HER2+ breast cancer cells to trastuzumab and lapatinib, we performed a high-throughput screen of 810 miRNA mimics and 816 miRNA inhibitors in KPL4 and SUM190PT cell lines treated with either of the drugs or the combination. The cell lines were chosen due to their low response to the two drugs.

**Figure 1:**
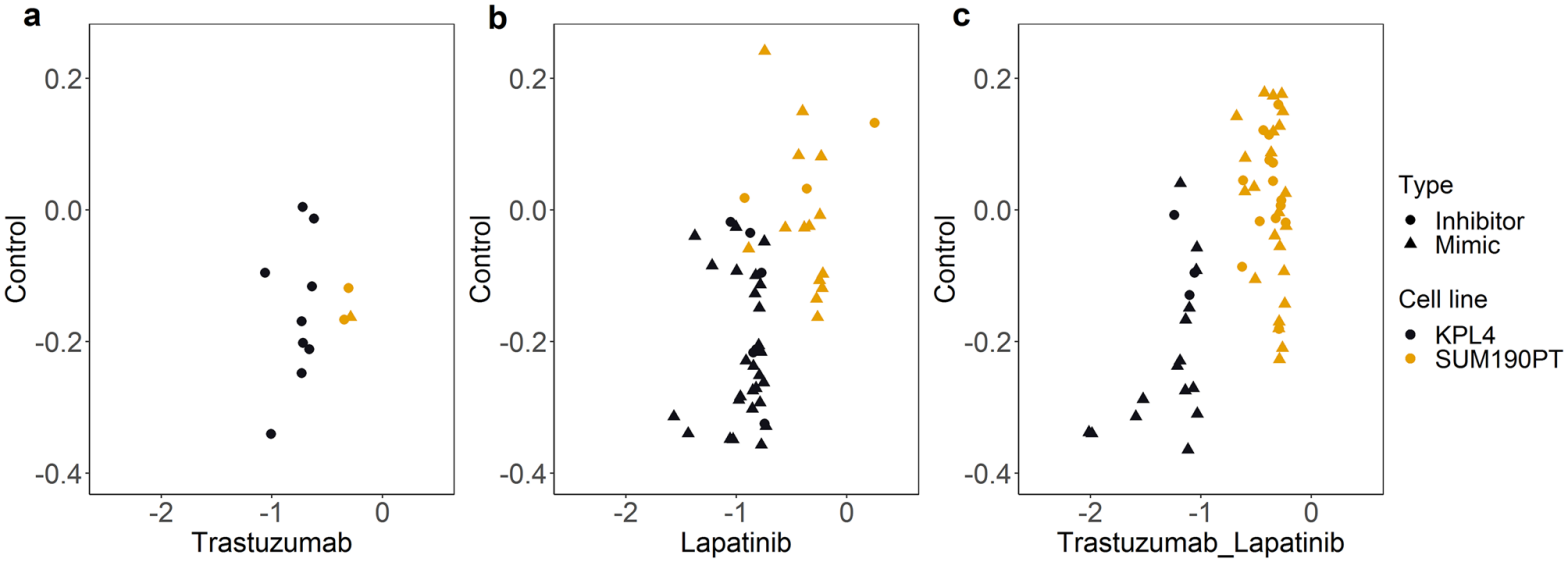
Viability for sensitizing miRNAs in high-throughput screen. The cells were treated with (**a**) Trastuzumab (10 μg/mL), (**b**) Lapatinib (100 nM) or (**c**) Trastuzumab and lapatinib together with a panel of miRNAs, and viability was measured using a CellTiter-Glo assay after 72 h. The scatterplots show viability (loess log) of treated cells (x-axis) vs untreated cells (y-axis) for significant miRNAs. I.e. miRNAs causing a viability reduction greater than three standard deviations from the median of all miRNAs in the same treatment group. Each point represents either a miRNA inhibitor (dot) or a miRNA mimic (triangle). Black color indicates result in KPL4 cells, and orange color indicates result in SUM190PT cells.

Of the 1626 miRNA mimics and inhibitors, 93 significantly affected the cell viability in one or more treatment groups compared to the control group (vehicle treated cells; Supplementary Table S5). The miRNA sensitization effects were dependent upon the treatment (Fig. 1). There were few sensitization effects with only trastuzumab treatment (Fig. 1a) and more in the lapatinib (Fig. 1b) and combination treated cells (Fig. 1c). In addition, the miRNA inhibitors were accountable for all but one sensitizing case in the trastuzumab setting (Fig. 1a), while the mimics were the sensitizing agent in the majority of cases in the lapatinib (Fig. 1b) and lapatinib plus trastuzumab (Fig. 1c) treated cells. Overall, there was a stronger effect of miRNAs on cell viability in KPL4 than in SUM190PT cells.

Sixteen miRNA candidates were selected for further exploration. The selection was based on miRNAs having multiple sensitization effects, either with an effect in both cell lines, or in more than one treatment setting within one cell line, e.g. sensitizing for both lapatinib and for the combination of trastuzumab and lapatinib. These candidates included two inhibitors: miR-361-5p and miR-502-3p; and fourteen mimics: mir-15b-5p, miR-19b-2-5p, miR-26b-3p, miR-29a-3p, miR-29c-3p, miR-32-3p, miR-93-3p, miR-101-5p, miR-106a-5p, miR-132-3p , miR-153-3p, miR-518a-5p, miR-744-3p and miR-1237-3p. The miRNA mimics and inhibitors caused little effect on cell viability alone in the screen, but in the presence of lapatinib and/or trastuzumab the viability decreased (Supplementary Table S5).

### Protein expression changes after miRNA and HER2-targeted treatment

In order to investigate possible pathways that could be affected by the miRNA mimics and inhibitors and HER2-targeted treatment, the two cell lines were treated with the drugs and subjected to a second miRNA screen. Cell lysates were prepared directly in the wells, and selected protein markers representing apoptosis, cell viability and members of the HER2 pathway were measured using a lysate microarray method (LMA). Protein deregulations with Z score ± 2 standard deviations were considered hits. We used the cell viability data to select miRNAs from the screen (*n* = 16), and the protein deregulations for these miRNAs in KPL4 cells are shown in Supplementary Table S6. Overall, the miRNAs showed most effect on protein expression levels in cells treated with lapatinib alone or in combination with trastuzumab, indicating that lapatinib was the strongest influencer. This was in concordance with the viability data. The protein data suggested miRNA-associated downregulation of proliferative markers and pathways (KI67, ERK, AKT and HER2), and upregulation of the apoptotic marker cPARP (Supplementary Table S6). This is in line with the decreased cell viability observed upon miRNA and HER2-targeted treatment.

### Eight miRNAs sensitize KPL4 cells to HER2-targeted treatment

**Figure 2:**
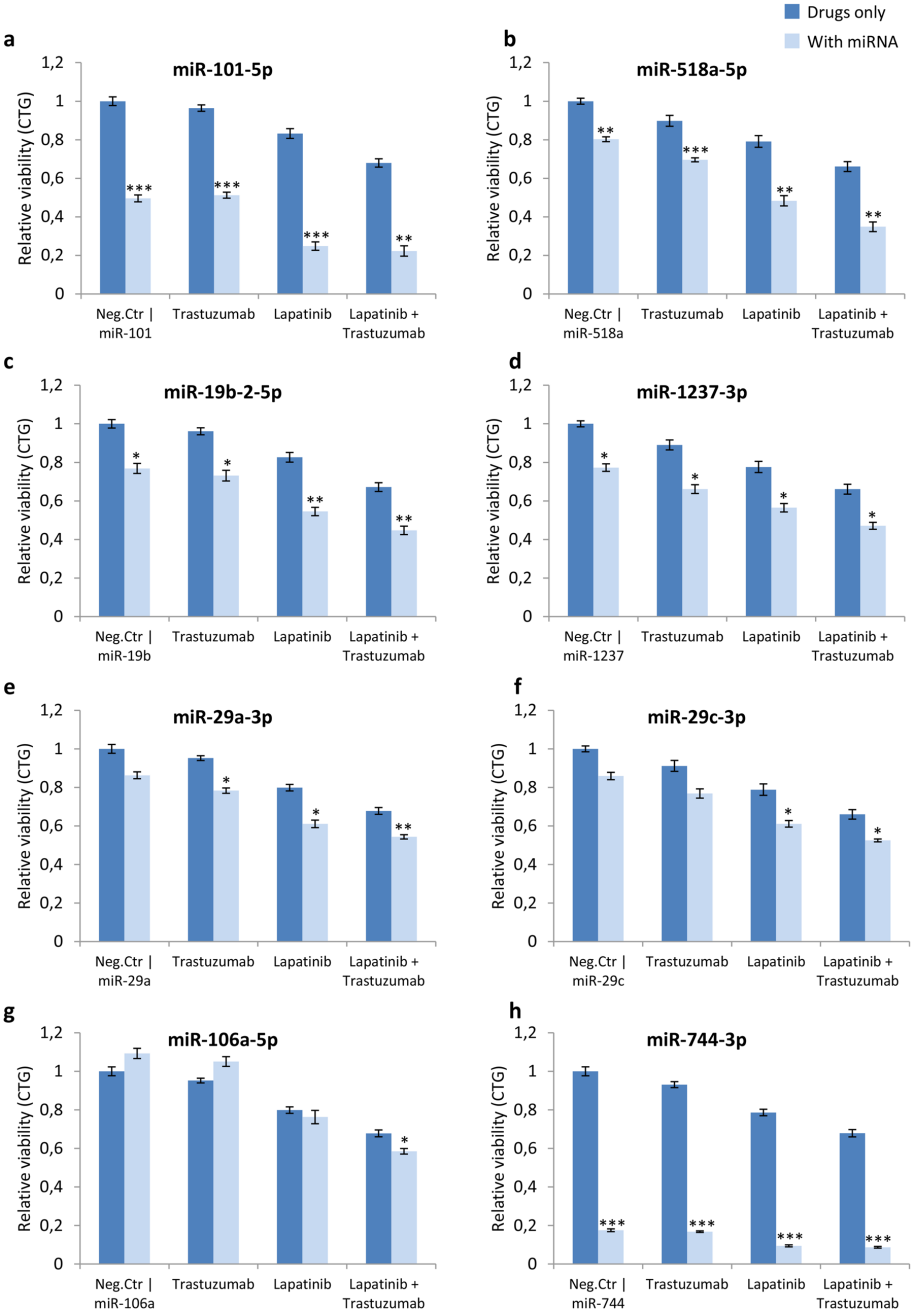
Viability measurements after treatment with sensitizing miRNAs. Viability measurements (by CellTiter-Glo) in cells transfected with indicated miRNA and treated with trastuzumab (10 μg/mL) and lapatinib (100 nM). The figure presents the top eight miRNA mimics that significantly sensitize KPL4 cells to trastuzumab and / or lapatinib. Viability is normalized to the median negative control for each plate. Each experiment is performed with three technical replicates and three biological replicates. Error bars = SEM. *p < *0.05*, **p < 0.01, ***p < 0.001 (Student’s T-test).

Next, we validated the 16 most promising sensitization candidates by a cell viability assay using KPL4 cells. This was the cell line presenting the strongest hits in the original screen. The miRNA mimics and inhibitors were transfected into the cells simultaneously as the cells were treated with trastuzumab and/or lapatinib. CellTiter-Glo assay was used to measure viability after incubation. Of the 16 miRNA mimics and inhibitors, 8 miRNA mimics showed a significant reduction in viability, compared to treatment with the scrambled negative control, in combination with either trastuzumab, lapatinib or both drugs (Student’s T-test, *p*-value < 0.05). These eight miRNA mimics were miR-101-5p, mir-518a-5p, miR-19b-2-5p, miR-1237-3p, miR-29a-3p, miR-29c-3p, miR-106a-5p, and miR-744-3p (Fig. 2).

The effect of the HER2-targeting drugs alone in KPL4 cells was a 4% reduction in viability due to trastuzumab, while lapatinib lowered the viability with 17% and finally, the combination of lapatinib and trastuzumab resulted in a 33% viability reduction compared to untreated cell. Addition of the above-mentioned eight miRNAs to the targeted treatment further reduced the viability to various extents (Fig. 2). miR-101-5p alone reduced cell viability with 50% (*p* < 0.001), and in combination with lapatinib, the viability was reduced with 75% (*p* < 0.001; Fig. 2a). The other seven miRNA mimics, miR-518a-5p, miR-19b-2-5p, miR-1237-3p, miR-29a-3p, miR-29c-3p, miR-106a-5p, mir-744-3p, all significantly reduced cell viability either alone or in combination with the two HER2-targeting drugs compared to the drugs alone (Fig. 2). The data from these eight mimics provide evidence that transfection of a single miRNA can affect cell viability and improve the in vitro efficacy of traditional drugs.

We evaluated the intrinsic expression of these eight sensitizing miRNAs in the previously described poorly responsive cell lines (KPL4 and SUM190PT) and the two responsive cell lines (SKBR3 and BT-474) before and after treatment with trastuzumab and/or lapatinib. The expression of the miRNAs did not significantly change due to HER2-targeted treatment. Further, there was no significant difference between the expression of the miRNAs in untreated responsive or untreated poorly responsive cells (data not shown).

### Correlations between miRNAs and mRNAs in primary tumors

To elucidate the clinical relevance of the sensitizing miRNAs, we calculated for each of the miRNAs the Spearman correlation between its expression and all mRNAs across 377 primary tumors. In this analysis miRNA expression was available for six out of the eight sensitizing miRNAs. To get an overall insight into biological processes associated with the sensitizing miRNAs, both positive and negative correlations were considered. The number of significant correlations varied from 86 (miR-1237) to 5943 mRNAs (miR-29c) (Supplementary Table S7), and the correlation coefficients were almost equally distributed into positive (52.6%) and negative (47.4%). For each miRNA, the list of significantly correlated mRNAs was imported to the Enrichr online tool for pathway enrichment analyses^21,22^ and KEGG 2019 Human pathways with an adjusted *p*-value below 0.05 were considered significant.

For miR-29a and miR-29c, we identified fifteen and two correlating pathways, respectively. For both miRNAs, cell cycle was the most significant reported pathway from the enrichment analyses (*p* = 1.05*10^–15^, and *p* = 3.94*10^–6^, respectively; Supplementary Table S7). Resembling pathway results for miR-29a and miR-29c is not surprising, as 26% of the significantly correlated mRNAs of these miRNAs overlapped. DNA replication and p53 signaling pathway were also among the significant pathways enriched among the correlated mRNAs of mir-29a. There were no significant pathways enriched among the genes correlated with miR-101, miR-1237, miR-518a and miR-744. Without *p*-value correction, MAPK signaling was among the significant pathways for miR-1237 (*p* = 0.04). These miRNA-mRNA correlations and pathway analyses may help explain some of the molecular mechanisms associated with the sensitizing miRNAs.

**Figure 3:**
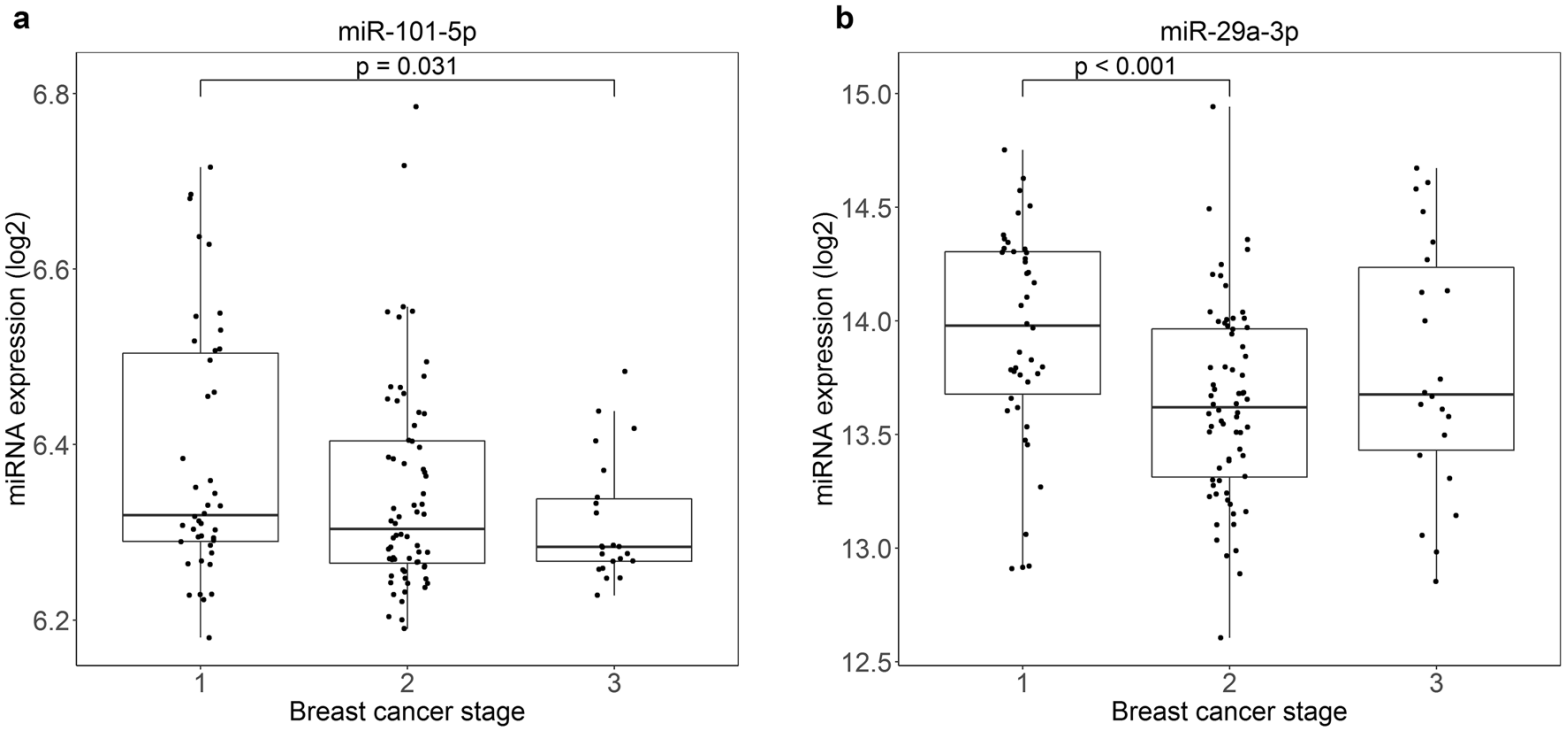
Expression of miR-101-5p and miR-29a-3p decrease with advanced breast cancer stage. miRNA expression and breast cancer stage in HER2+ breast cancer patients from the METABRIC dataset. The boxplots show miRNA expression level on the y-axis and breast cancer stage on the x-axis. There is a significantly higher expression of miR-101-5p in stage 1 patients versus stage 3 patients (*p* = 0.031; (**a**)) and of miR-29a-3p in stage 1 patients versus stage 3 patients (*p* < 0.001; (**b**)). *p*-values resulting from Wilcoxon tests.

We further studied the association between HER2+ tumor miRNA expression and breast cancer stage in the Molecular Taxonomy of Breast Cancer International Consortium (METABRIC) dataset, where four of the eight miRNAs were expressed (mir-101-5p, miR-29a-3p, miR-29c-3p and miR-744-3p). There was a higher expression of miR-101-5p and miR-29a-3p in the early stage group compared to the more advanced stages of breast cancer (Fig. 3). Specifically, there was a significant difference between breast cancer stages 1 and 3 for miR-101-5p expression (*p* = 0.031), and between stages 1 and 2 for miR-29a-3p (*p* < 0.001). These results further support a tumor suppressor role for the two miRNAs.

### miR-101-5p expression is associated with survival in HER2 + patients

**Figure 4:**
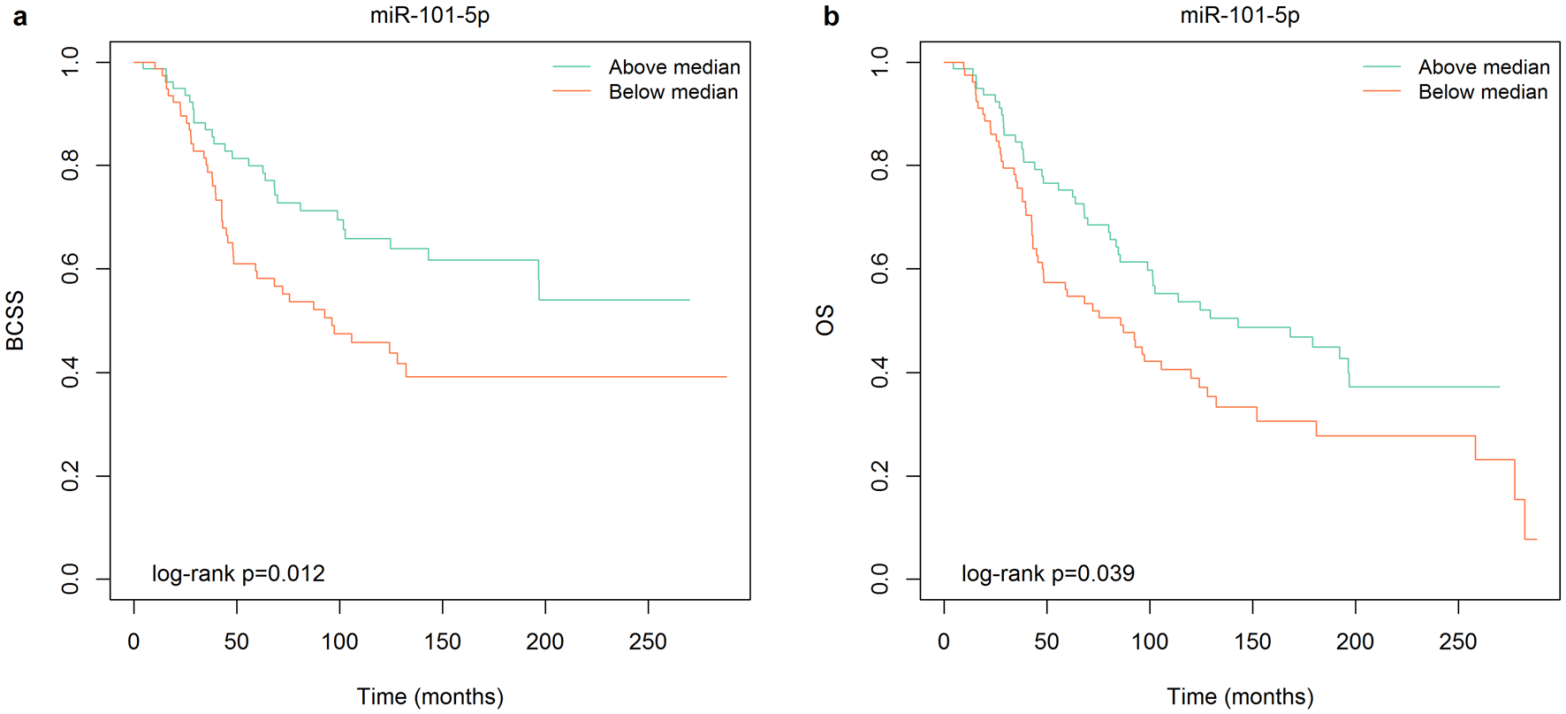
Higher expression of miR-101-5p is associated with better survival of HER2 + breast cancer patients. Survival curves presented as Kaplan–Meier plots for HER2 + breast cancer patients in the METABRIC dataset (*n* = 160). There is a significantly better breast cancer specific survival (BCSS; *p* = 0.012; (**a**)) and overall survival (OS; *p* = 0.039; (**b**)) with above median expression of miR-101-5p.

Next, we investigated miRNA expression in relation to breast cancer specific survival (BCSS) and overall survival (OS) in HER2 + patients from the METABRIC and The Cancer Genome Atlas (TCGA) datasets. Of note, data for METABRIC was collected before trastuzumab was introduced, thus the patients had not received HER2-targeted therapy. Patients with a high expression (> median) of miR-101-5p had a significantly higher BCSS and OS in METABRIC (BCSS: *p* = 0.012, OS: *p* = 0.039; Fig. 4). No significant difference was found within the TCGA dataset. Kaplan–Meier plots for the other miRNAs can be found in Supplementary Fig. S3. This suggests that miR-101-5p expression has prognostic value for HER2 + breast cancer patients. In addition, it supports our finding of miR-101-5p being a favorable miRNA with tumor suppressor properties in HER2 + breast cancer.

## Discussion

Although targeted therapeutics have improved survival for HER2-positive breast cancer patients, poor response and disease progression is still a major problem. The treatment is continuously evolving, including agents as trastuzumab, lapatinib and pertuzumab, to the lately approved tucatinib and margetuximab-cmkb. These drugs are often administered in combination with chemotherapy. In this study, we present miRNAs that sensitize HER2-positive breast cancer cells to HER2-targeted treatment.

First, we explored miRNA expression changes in breast cancer cell lines with different response to trastuzumab and lapatinib. Based on the expression profiles before and after treatment, we saw that responsive cell lines had a higher number of deregulated miRNAs (*n* = 36) than less responsive cell lines. This indicates that the miRNAs altered in expression may be associated with some of the molecular mechanisms contributing to reduced cell viability in the responsive cell lines. The predicted mRNA targets of the miRNAs in the responsive cell lines were involved in the MAPK signaling pathway, which can explain some of the response mechanisms upon treatment.

The effect of trastuzumab and lapatinib on the endogenous miRNA expression landscape showed differences between responsive and poorly responsive cell lines. This was however not sufficient to identify miRNAs sensitizing HER2 + breast cancer cells to targeted treatment. In order to identify such miRNAs and to look into possible mechanisms involved, we performed a high-throughput screen and used cell viability and expression of central growth regulatory and apoptotic proteins as readout. Through validation in vitro*,* we report eight miRNAs as sensitizing agents that downregulate cell viability in KPL4 cells alone or in combination with trastuzumab and/or lapatinib.

The protein lysate analyses included proteins that represents proliferation and growth (KI67, HER2, phosphorylated HER2, ERK and total AKT) and apoptosis (cPARP). The data showed deregulations that may explain some of the reduction in viability we observe upon miRNA and targeted treatment. miR-101-5p in combination with trastuzumab and lapatinib downregulated HER2, p-HER2-Y1222 and ERK, which supports the decrease in cell viability (Supplementary Table S6). When affecting targets in the HER2 axis, the cells’ ability to survive is weakened. For the seven other validated miRNAs presented here, all significant protein deregulations from the initial screen represented changes that support the tumor suppressive role of these miRNAs. Downregulation of one or more proliferative markers was observed for five miRNAs (miR-101-5p, miR-19b, miR-29a, miR-518a and miR-744), and upregulation of the apoptotic marker cPARP was observed for one miRNA (miR-744).

We here present the growth inhibitory and sensitizing effects of miR-101-5p in HER2-positive breast cancer cells. This miRNA in combination with lapatinib caused a pronounced reduction in KPL4 cell viability and is a strong sensitizing candidate among the validated miRNAs. The growth inhibitory effects of this miRNA are supported by Toda and colleagues, who present miR-101-5p as a tumor suppressive miRNA regulating multiple oncogenic targets^23^. In addition, miR-101 has been demonstrated to promote apoptosis^24^ and inhibit growth in both in vitro and in vivo breast cancer models^25^. Furthermore, paclitaxel sensitivity increased through miR-101 by inhibiting MCL-1 in triple negative breast cancer^25^. Altogether, this may explain some of the growth inhibiting effect miR-101-5p exerts in KPL4 cells. To our knowledge, we are the first to report that miR-101-5p sensitizes HER2 + breast cancer cells to targeted treatment.

Our cell viability findings also suggest miR-19b to act as a tumor suppressor in KPL4 cells. Others have reported an opposite effect; the same miRNA promotes migration and invasion in ovarian cancer through the inhibition of PTEN^26^. However, as KPL4 cells are PTEN deficient^20^, other properties of the miRNA may be more dominant in our model system. Further, Li and colleagues report increased cell proliferation in MCF-7 and MDA-MB-231 cells when overexpressing miR-19b, which is contradictory to our results^27^. However, both these cell lines are HER2-negative. These opposing results clearly display the importance of cancer entity and cell line specificity when investigating the effect of biological modifiers, as for instance miRNAs. In a broader sense these observations underline the importance of personalized medicine.

In our study, miR-29a shows a growth inhibitory effect in KPL4 cells in combination with targeted treatment. This is supported in a study where overexpression of miR-29a inhibited breast cancer cell growth in MDA-MB-453 (HER2 +) and MCF-10A (normal epithelial breast) cells^28^. In pancreatic cancer cells, mir-29a functions as a tumor suppressor by targeting MUC1^29^. However, studies in breast cancer models also report miR-29a to contribute to epithelial to mesenchymal transition, migration and invasion in MDA-MB-231 (HER2-) and MCF-7 (HER2-) cells^30^, to play a role in drug resistance^31^, and to induce cell proliferation and metastasis in MCF-7 and T47D (HER2-) cells^32^. Once again, the diverging effects of specific miRNAs must be considered in the context of the specific cell line and model system.

We show that another member of the miR-29 family, miR-29c, exerts similar effects on KPL4 cell viability as miR-29a. Work by Li and colleagues reflects the favorable properties of miR-29c in breast cancer by regulating the TIMP/STAT1/FOXO1 pathway^33^. We have previously described mir-29c to target the immunoregulatory protein B7-H3, and the expression of miR-29c is associated with survival in breast cancer patients^12^. These data substantiate miR-29c as a tumor suppressor.

miR-518a, miR-106a and miR-1237 all increase the sensitivity of KPL4 cells to HER2-targeting drugs. The tumor suppressing or growth inhibitory roles of these miRNAs are supported in various cancer forms. miR-518 is reported to be favorable in colorectal^34^, gastrointestinal stromal^35^ and oral squamous cell carcinoma^36^. In astrocytoma cells, miR-106a-5p is reported to inhibit proliferation and promote apoptosis^37^, which supports the growth inhibitory role of this miRNA. A reduced expression level of miR-1237 was associated with tumor invasion and worse recurrence-free survival in spinal chordoma patients^38^. Despite different model systems and cancer types, these studies substantiate our results showing that upregulation of these miRNAs is favorable.

We have previously reported miR-744 as significantly downregulated in HER2-positive breast cancer tumors compared to HER2-negative tumors in two clinical cohorts^7^. When overexpressing this miRNA in KPL4 cells it reduced the cell viability with 82%. Although the miRNA increases tumorigenicity and progression in pancreatic^39^, prostate^40^ and laryngeal squamous cell carcinoma^41^, the opposite effect is reported in ovarian^42^, cervical^43^ and hepatocellular carcinoma^44^ in addition to breast cancer^45^. When we stratify for HER2-positive breast cancer patients, there is not a significant correlation between high mir-744 expression and survival in the METABRIC or TCGA datasets. But across all breast tumors; ER/PR-positive; lymph node-negative or non-metastatic disease, Kim et al. report there is an association between high mir-744 expression and increased survival^46^, which supports the role of miR-744 as a tumor suppressor in breast cancer.

The correlation analyses between the sensitizing miRNAs and mRNAs in a clinical cohort resulted in 9840 significant miRNA-mRNA correlations. About half of the correlations were positive, underlining that this analysis captures an indirect as well as direct relationship between miRNAs and mRNAs. The association between both miR-29a and miR-29c and their correlated mRNAs to cell progression through cell cycle signaling may contribute to deciphering the sensitizing effect of these miRNAs.

Of the sensitizing miRNAs presented here, both miR-101-5p and miR-29a-3p show higher expression levels in early stage versus more advanced stage breast cancers among HER2 + patients in the METABRIC cohort. This supports that high expression levels are favorable. Further, miR-101-5p correlates significantly to survival in HER2 + patients in the METABRIC dataset, where a high expression is associated with favorable outcome. This has also been shown in non-small cell lung cancer^47^. Based on the survival analysis combined with the in vitro viability data and the link to early breast cancer stage presented here, miR-101-5p is a strong candidate to sensitize HER2-positive breast cancer cells to targeted treatment.

To conclude, the combination of sensitizing miRNAs and targeted treatment has the potential to improve the current treatment for HER2 + breast cancer patients. We have identified eight miRNA mimics that downregulate cell viability in HER2 + cells in combination with lapatinib and/or trastuzumab. Of these, miR-101-5p shows the most promising growth inhibitory effect in combination with lapatinib. Clinical data confirm expression of miR-101-5p as favorable for breast cancer stage, disease specific survival and overall survival, which substantiates the relevance of this miRNA for HER2 + breast cancer.

## Methods

### Cell cultures

Four HER2 + human breast cancer cell lines were used in this study. KPL4 cells were provided by Professor J. Kurebayashi (Kawasaki Medical School, Japan)^48^. SUM190PT cells were provided from Karmanos Cancer Institute in Michigan, USA. BT-474 and SKBR3 cells were obtained from American Type Culture Collection (ATCC, Manassas, VA, USA). The growth media are described in Supplementary Table S1. Cells were cultured in 37 °C, 5% CO_2_ and for a maximum of 30 passages prior to use.

The cell lines were chosen based on their responsiveness to trastuzumab and lapatinib. Both KPL4 and SUM190PT have previously been found nonresponsive to trastuzumab, while SKBR3 and BT-474 cause > 20% growth inhibition^20^. KPL4 cells are less responsive to lapatinib (< 50% growth inhibition when cultured with 7 dilutions between 0.34 pM and 20 µM for 5 days), SUM190PT and BT-474 cells are intermediately responsive to lapatinib (60-65% growth inhibition under the same conditions) while SKBR3 is characterized as highly responsive (> 75% growth inhibition)^20^.

A summarizing flowchart that illustrates all parts of the project is presented in Supplementary Fig. S1.

### miRNA expression in HER2 + cell lines

KPL4, SUM190PT, SKBR3 and BT-474 cells were cultured in 6-well plates. After 20 h, 10 µg/mL trastuzumab (Roche Applied Biosciences, Basel, Switzerland), 100 nM Lapatinib Ditosylate (GW-572016, Selleckchem, Houston, TX, USA) or a combination of trastuzumab and lapatinib were added. After drug exposure for 24 h, the cells were scraped and RNA was purified using the miRNeasy Mini kit (Qiagen, Hilden, Germany) according to the manufacturer's protocols. RNA yield was assessed spectrophotometrically (NanoDrop 2000, Thermo Fisher Scientific, Waltham, MA USA). Expression of miRNA was measured by the one-color microarray SurePrint Human miRNA Microarray with design ID 031181, release 16.0, 8 × 60 K (Agilent Technologies, Santa Clara, CA, USA) according to the protocol supplied by the manufacturer (miRNA Microarray System v2.3). Scanning was performed on Agilent Scanner G2565B. Samples were processed using Feature Extraction (FE) version 10.7.3.1 (Agilent Technologies). Quality was assessed by the quality control parameters in FE. The data were log2-transformed and for each sample, considering only expressed miRNAs, the data were median centered. All non-expressed miRNAs across samples were set to a common minimum value. miRNA expression data are available in the Gene Expression Omnibus database^49^ (https://www.ncbi.nlm.nih.gov/geo/) under accession number GSE163490.

Kruskal–Wallis tests performed in R version 4.0.2 in R Studio version 1.1.423^50^, were used to study deregulations in miRNA expression levels before and after drug treatment with trastuzumab and lapatinib. The miRNAs were tested based on the cell lines' response to trastuzumab and lapatinib in three groups (changes in expression before vs. after drug treatment): all four cell lines combined (SKBR3, BT-474, KPL4 and SUM190PT), responding cell lines (SKBR3 and BT-474), and poorly responding cell lines (KPL4 and SUM190PT). miRNA expression level changes with a nominal *p*-value < 0.05 were considered statistically significant.

### MiRNA target prediction and enrichment analyses

To identify in silico predicted mRNA targets of regulated miRNAs, we used IPA (QIAGEN Inc., https://www.qiagenbioinformatics.com/products/ingenuity-pathway-analysis^51^ version 57662101. The MicroRNA Target Filter was used to predict mRNA targets and we selected ‘Experimentally observed’ and ‘High prediction’ confidence targets. The sources of the predictions within the tool were Ingenuity Expert Findings, Ingenuity ExpertAssist Findings, TargetScan Human^52^, miRecords^53^ and TarBase^54^. Enrichment analyses were performed in Enrichr^21,22^, where KEGG 2019 Human pathways with an adjusted *p*-value below 0.05 were considered significant.

### High-throughput miRNA screen and HER2-targeted treatment

miRIDIAN miRNA Human Mimic Library and miRIDIAN miRNA Inhibitor Library (v. 10.1; Dharmacon, Lafayette, CO, USA) were used to transfect KPL4 and SUM190PT cells with 810 miRNA mimics and 816 miRNA inhibitors. Clear polystyrene 384 well microplates (Sigma Aldrich, St. Louis, MO, USA) were pre-printed with the miRNA libraries to achieve a final concentration of 20 nM. SiLentFect™ Lipid Reagent for RNAi (Bio-Rad Laboratories, Hercules, CA, USA) diluted in OptiMEM (Gibco Invitrogen, Carlsbad, CA) was used for transfection. Cells were seeded in the wells (2000 cells/well) and treated with vehicle, 10 µg/mL trastuzumab (Roche Applied Biosciences), 100 nM Lapatinib Ditosylate (GW-572016, Selleckchem) or a combination of trastuzumab and lapatinib. AllStars Hs Cell Death Control siRNA (Qiagen, Chatsworth, CA, USA) was used as a positive control and miRIDIAN microRNA Mimic Negative Control #1, miRIDIAN microRNA Mimic Negative Control #2, miRIDIAN microRNA Hairpin Inhibitor Negative Control #1 (Dharmacon), Negative Control siRNA, miScript Inhibitor Negative Control, AllStars Negative Control siRNA (Qiagen), anti-miR negative control #1, pre-miR negative control #1, and pre-miR negative control #2 (Ambion Inc., Austin, TX, USA) were used as negative controls. The cells were incubated for 72 h at 37 °C, 5% CO_2_.

### Cell viability following miRNA transfection

Cell viability was measured using CellTiter-Glo Luminescent Cell Viability Assay (Promega, Madison, WA, USA) according to provider's protocol, and output was measured on the Envision plate reader (PerkinElmer, Norwalk, CT, USA). The data were Loess normalized^55^ and log2-transformed.

To determine which miRNAs were sensitizing the cells to treatment, we considered the cell viability inhibition of all miRNAs within the same treatment group (i.e. no drug, lapatinib, trastuzumab or lapatinib plus trastuzumab). MiRNAs resulting in a viability inhibition larger than three standard deviations (SD) from the median of all miRNAs within the same treatment group were considered sensitizing. Two exclusion criteria were then applied: firstly, the miRNAs should have no significant effect (SD < 3) on viability in cells without drug treatment, and secondly, miRNA inhibitors targeting miRNAs not endogenously expressed in the untreated cells were excluded.

### Protein expression analysis by lysate microarrays

A second set of plates with miRNA and drug treated cells were prepared identically to run protein analyses using an LMA method, as previously described^8^. In brief, after the 72 h incubation period, the cells were lysed directly in the 384 well plates using 15 µl of lysis buffer (100 mM Tris, pH 8.0; 0.2% SDS; 25 mM DTT) before denaturation at 95 °C for 15 min. The lysates were printed on nitrocellulose-coated microarray slides (FAST™ slides, Whatman Inc., Florham Park, NJ, USA) and blocked with near-infrared blocking buffer (Rockland Immunochemicals, Inc., Gilbertsville, PA, USA). Antibodies for AKT, pAKT, ERK, and pERK (Cell Signaling Technology Inc., Danvers, MA, USA), cPARP (Abcam, Cambridge, UK), KI67, HER2 (Dako, Glostrup, Denmark), pHER2-Y1222 and pHER2-Y877 (Cell Signaling Technology Inc.) were used, in addition to Sypro Ruby Blot solution (Invitrogen Inc., Carlsbad, CA, USA) for total protein measurement. The Sypro signal was detected using Tecan LS400 (Tecan Inc., Durham, NC, USA) microarray scanner at 488/670 nm, and fluorescence from the secondary antibodies was detected at 700 nm using Odyssey Licor IR-scanner (LI-COR Biosciences, Lincoln, NE, USA). To measure the median pixel intensities of each spot and the slide background from each channel Array-Pro Analyzer Microarray Analysis Software (Median Cybernetics Inc., Bethesda, MD) was used. The data were normalized to the Sypro signal, log2-transformed and Z-score normalized.

### Validation of miRNAs sensitizing single and combinatorial HER2-targeted treatment

For the validation of miRNAs showing sensitization effect on viability, Costar 96-well white, clear-bottom polystyrene plates (Corning Inc., Corning, NY, USA) were used. Sixteen miRNAs (14 mimics: hsa-mir-15b-5p, hsa-miR-19b-2-5p, hsa-miR-26b-3p, hsa-miR-29a-3p, hsa-miR-29c-3p, hsa-miR-32-3p, hsa-miR-93-3p, hsa-miR-101-5p, hsa-miR-106a-5p, hsa-miR-132-3p, hsa-miR-153-3p, hsa-mir-518a-5p, hsa-miR-744-3p , hsa-miR-1237-3p, two inhibitors: hsa-miR-361-5p and miR-502-3p, and miRIDIAN Mimic Negative Control #1, (Dharmacon)) and AllStars HS Cell Death Control siRNA (Qiagen) were added to the wells at a final concentration of 20 nM. SiLentFect™ Lipid Reagent (Bio-Rad Laboratories) was diluted in Opti-MEM™ Reduced Serum Medium, no phenol red (Thermo Fisher Scientific), incubated for 10 min at room temperature and added to the wells. The miRNAs/siRNAs and the lipids were incubated for 1 h at room temperature. Thereafter, 10,000 KPL4 cells were added to each well in combination with 100 nM lapatinib (dissolved in DMSO and diluted in cell culture medium) (Selleckchem), 10 µg/mL trastuzumab (Roche Applied Biosciences) or the combination of the two drugs in the same concentrations. All wells contained 0.0023% DMSO as lapatinib was dissolved in DMSO. The plate was incubated for 72 h at 37 °C, 5% CO_2_. Cell viability was measured using CellTiter-Glo Assay and Victor X Plate Reader (PerkinElmer). The raw data were normalized to the negative control median plate wise. All experiments were conducted in three parallels with three technical replicates. *P*-values were calculated using a Student’s t-test.

### Validation in clinical cohorts

Expression data from miRNA and mRNA of primary tumors from 377 breast cancer patients in the Oslo2 study were used for correlation analyses. The Oslo2 study is approved by Regional committees for medical and health research ethics (approval number 2016/433; 429-04148), with informed consent obtained from all participants and/or their legal guardians^56,57^. All research was performed in accordance with relevant guidelines and regulations. miR-106a-5p was not present in the clinical dataset. miR-19b-2-5p was present, but not expressed in the Oslo2 dataset. Spearman correlations were performed in R (v. 4.0.2) in R Studio (v.1.1.423, RStudio Team). The correlation *p*-values were Benjamini–Hochberg corrected and considered significant for *p* < 0.05. Enrichr^21,22^ was used for enrichment analyses, and KEGG 2019 Human pathways with an adjusted *p*-value below 0.05 were considered significant*.* miRNA expression, breast cancer stage and survival data from breast cancer patients in the METABRIC cohort (*n*_HER2+_  = 160)^58^ and the TCGA dataset (*n*_HER2+_  = 69) were used for clinical analyses. We performed Wilcoxon tests to study the relationship between miRNA expression and breast cancer stage. Breast cancer stages 0 and 4 were excluded in the METABRIC dataset due to few patients (*n* = 4 and *n* = 1, respectively). There were too few patients with both miRNA expression and stage data in the TCGA dataset to perform analysis. For the survival data, both datasets were analysed. Here, a log-rank test was applied to test the difference between the Kaplan–Meier curves when patients were split into two groups based on miRNA expression below or above median expression.

## Supplementary Information

Supplementary Information 1.

Supplementary Information 2.

## Data Availability

miRNA expression data are available in the Gene Expression Omnibus database^49^ (https://www.ncbi.nlm.nih.gov/geo/) under accession number GSE163490. All other data generated or analysed during this study are included in this published article (and its Supplementary Information files) or publically available.
