# Supplementary material for: MicroRNA in combination with HER2-targeting drugs reduces breast cancer cell viability in vitro
Source: Sci Rep. 2021 May 25;11:10893. doi: 10.1038/s41598-021-90385-2 (PMC8149698; doi:10.1038/s41598-021-90385-2)
Supplement: Supplementary file 1 — Supplementary Information 1. [file 41598_2021_90385_MOESM1_ESM.zip › Suppl. Table S1.pdf]

MicroRNA in combination with HER2-targeting drugs reduces breast cancer cell viability *in vitro*

Lisa Svartdal Normann[1,2,3], Miriam Ragle Aure[3], Suvi-Katri Leivonen[4], Mads Haugland Haugen[2], Vesa Hongisto[5], Vessela N. Kristensen[3,6], Gunhild Mari Mælandsmo[2,7], and Kristine Kleivi Sahlberg[1,2].

Addresses

[1]Department of Research and Innovation, Vestre Viken Hospital Trust, Drammen, Norway.
[2]Department of Tumor Biology, Institute for Cancer Research, The Norwegian Radium Hospital, Oslo University Hospital, Oslo, Norway.
[3]Department of Medical Genetics, Institute of Clinical Medicine, Faculty of Medicine, University of Oslo, Oslo, Norway.
[4]Applied Tumor Genomics Research Program, Medical Faculty, University of Helsinki, Helsinki, Finland.
[5]Division of Toxicology, Misvik Biology, Turku, Finland.
[6]Department of Clinical Molecular Biology (EpiGen), Division of Medicine, Akershus University Hospital, Lørenskog, Norway.
[7]Institute for Medical Biology, Faculty of Health Sciences, UiT – The Arctic University of Norway.

Correspondence

Dr. K. K. Sahlberg,

Department of Research and Innovation, Vestre Viken Hospital Trust, P.O. Box 800, 3004 Drammen, Norway.

E-mail: Kristine.Sahlberg@vestreviken.no

Supplementary Table S1. Growth media for cell culturing.

Cell type	Experimental setup	Growth medium
KPL4	miRNA expression<br>miRNA screen	Dulbecco's Modified Eagle's Medium (4.5 g/L glucose) (DMEM) (Sigma–Aldrich (S-A), St. Louis, MO, USA) supplemented with 10 % fetal bovine serum (FBS), 2 mM L-glutamine and 1 % penicillin/Streptomycin (S-A).
SUM190PT	miRNA expression<br>miRNA screen	Ham's F-12 + Glutamax supplemented with 5 µg/mL insulin, 0.1 % hydrocortisone, 2.5 µg/mL fungizone, 25 µg/mL gentamicin, 2.5 µg/mL plasmocin, 5 mM ethanolamine, 10 mM Hepes, 5 µg/mL transferrin, 10 nM T3, 50 nM sodium selenite, and 1 g/L Bovine Serum Albumine (S-A). Immediately after plating 2 % of FBS was added to the medium for the cells to be able to attach. After 24h the medium was shifted to serum-free.
BT-474	miRNA expression	DMEM supplemented with 10 % FBS, 4 mM L-glutamine, 0.01 mg/ml insulin, 1 mM Na-pyruvate, and 1% penicillin/streptomycin (S-A).
SKBR3	miRNA expression	McCoy's 5A supplemented with 10 % FBS, 1.5 mM L-glutamine, and 1 % pen/strep (S-A).
KPL4	miRNA validation	DMEM supplemented with 10 % FBS, 1 % Glutamax and 2 % HEPES (S-A).
