# Supplementary material for: MicroRNA in combination with HER2-targeting drugs reduces breast cancer cell viability in vitro
Source: Sci Rep. 2021 May 25;11:10893. doi: 10.1038/s41598-021-90385-2 (PMC8149698; doi:10.1038/s41598-021-90385-2)

# MicroRNA in combination with HER2-targeting drugs reduces breast cancer cell viability *in vitro*

Lisa Svartdal Normann[1,2,3], Miriam Ragle Aure[3], Suvi-Katri Leivonen[4], Mads Haugland Haugen[2], Vesa Hongisto[5], Vessela N. Kristensen[3,6], Gunhild Mari Mælandsmo[2,7], and Kristine Kleivi Sahlberg[1,2].

**Addresses**

[1]Department of Research and Innovation, Vestre Viken Hospital Trust, Drammen, Norway.
[2]Department of Tumor Biology, Institute for Cancer Research, The Norwegian Radium Hospital, Oslo University Hospital, Oslo, Norway.
[3]Department of Medical Genetics, Institute of Clinical Medicine, Faculty of Medicine, University of Oslo, Oslo, Norway.
[4]Applied Tumor Genomics Research Program, Medical Faculty, University of Helsinki, Helsinki, Finland.
[5]Division of Toxicology, Misvik Biology, Turku, Finland.
[6]Department of Clinical Molecular Biology (EpiGen), Division of Medicine, Akershus University Hospital, Lørenskog, Norway.
[7]Institute for Medical Biology, Faculty of Health Sciences, UiT – The Arctic University of Norway.

**Correspondence**

Dr. K. K. Sahlberg,
Department of Research and Innovation, Vestre Viken Hospital Trust, P.O. Box 800, 3004 Drammen, Norway.
E-mail: Kristine.Sahlberg@vestreviken.no

Supplementary Figure S1



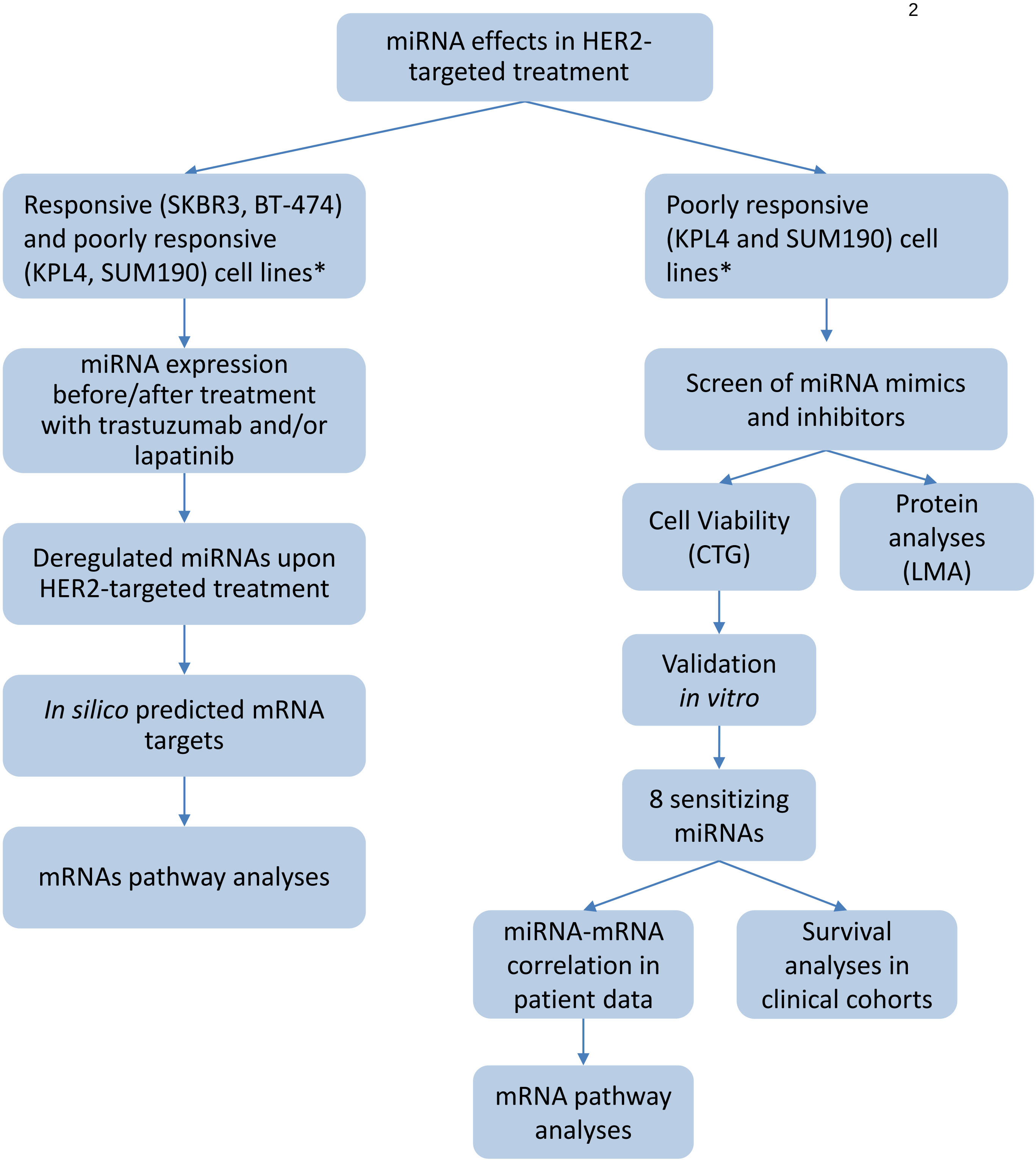


*Response to targeted treatment (trastuzumab and lapatinib).
CTG = CellTiter-Glo. LMA = Protein Lysate Microarray

**Supplementary Figure S1: Flowchart illustrating the main topics covered in the paper.** On the left side, the effects of HER2-targeted treatment on endogenous miRNA expression levels in HER2+ breast cancer cells are studied. On the right side, the effects of combinatorial treatment with HER2-targeted drugs and transfection of miRNAs in poorly responsive cell lines are explored.

# Supplementary Figure S2: miRNA expression before and after targeted treatment in four cell lines (SKBR3, BT-474, KPL4, SUM190PT)

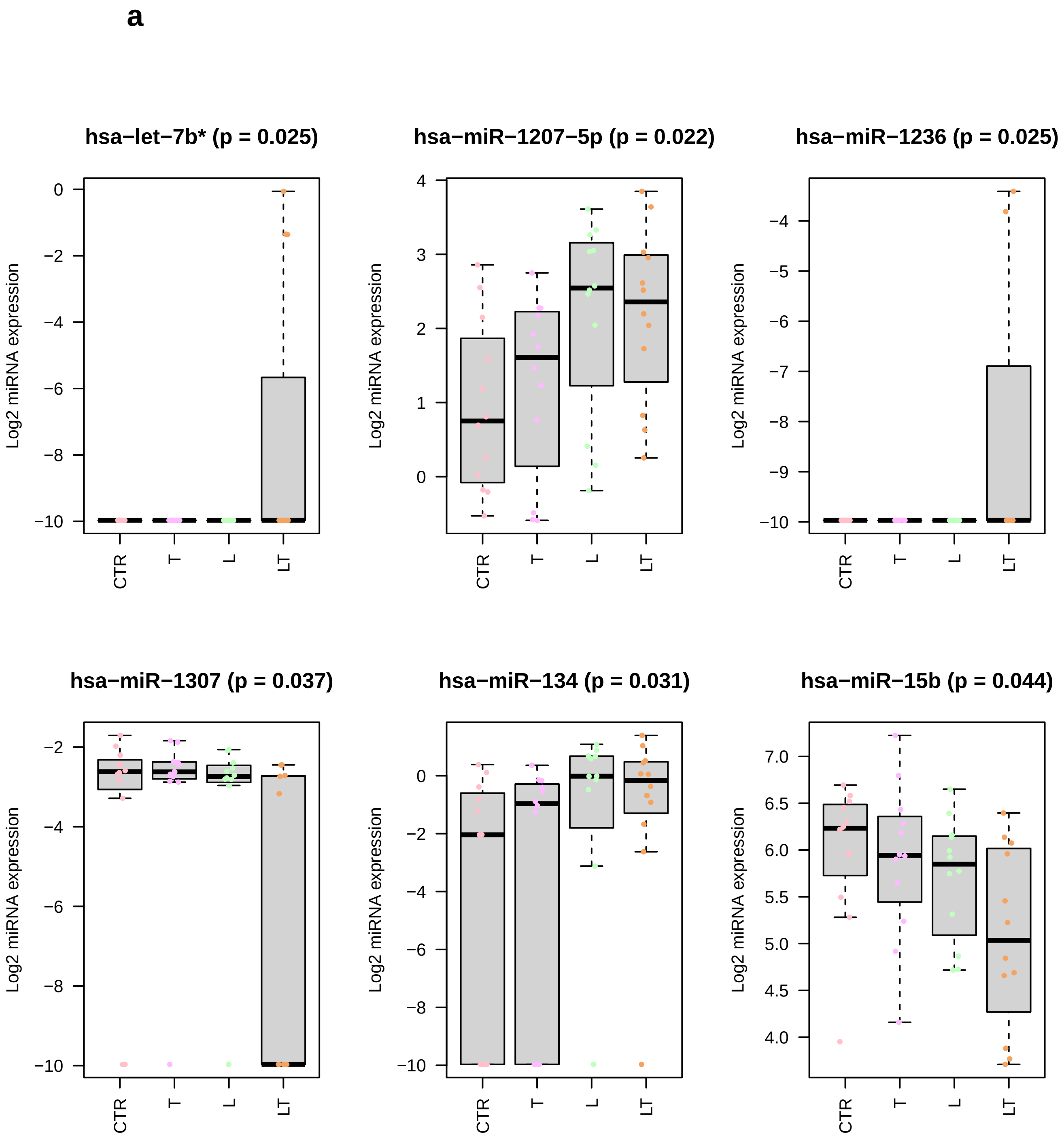


**Supplementary Figure S2: miRNA expression after targeted treatment.**
The cells were treated with trastuzumab (10 µg/mL) or lapatinib (100 nM) alone and in combination for 24 hours before total RNA was extracted, and miRNA levels were measured using a one-color microarray Human miRNA Microarray Kit and scanned on Agilent Scanner G2565B. miRNAs with significant (Kruskal-Wallis test p-value < 0.05) expression regulations between any treatment groups are presented. The plots are presented for a-b: four HER2+ cell lines pooled together (SKBR3, BT-474, KPL4, SUM190PT); c-h: cell lines responsive to trastuzumab and lapatinib (SKBR3 and BT-474); and i: poorly responsive cell lines (KPL4 and SUM190PT). T = trastuzumab, L = lapatinib, TL = trastuzumab and lapatinib.

Supplementary Figure S2: miRNA expression before and after targeted treatment in four cell lines (SKBR3, BT-474, KPL4, SUM190PT)

**b**

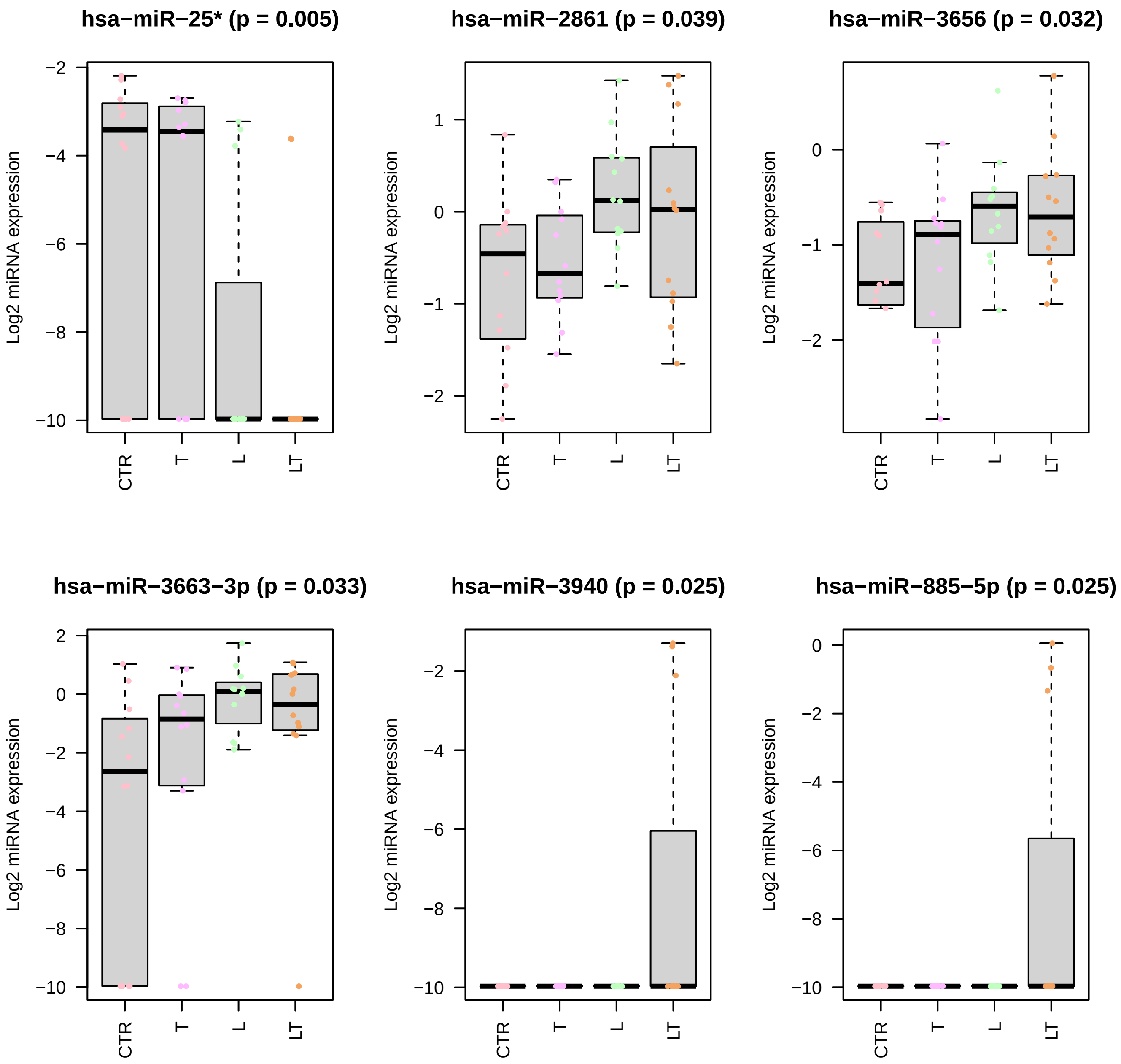

Supplementary Figure S2: miRNA expression before and after targeted treatment in responsive cell lines (SKBR3, BT-474)

c

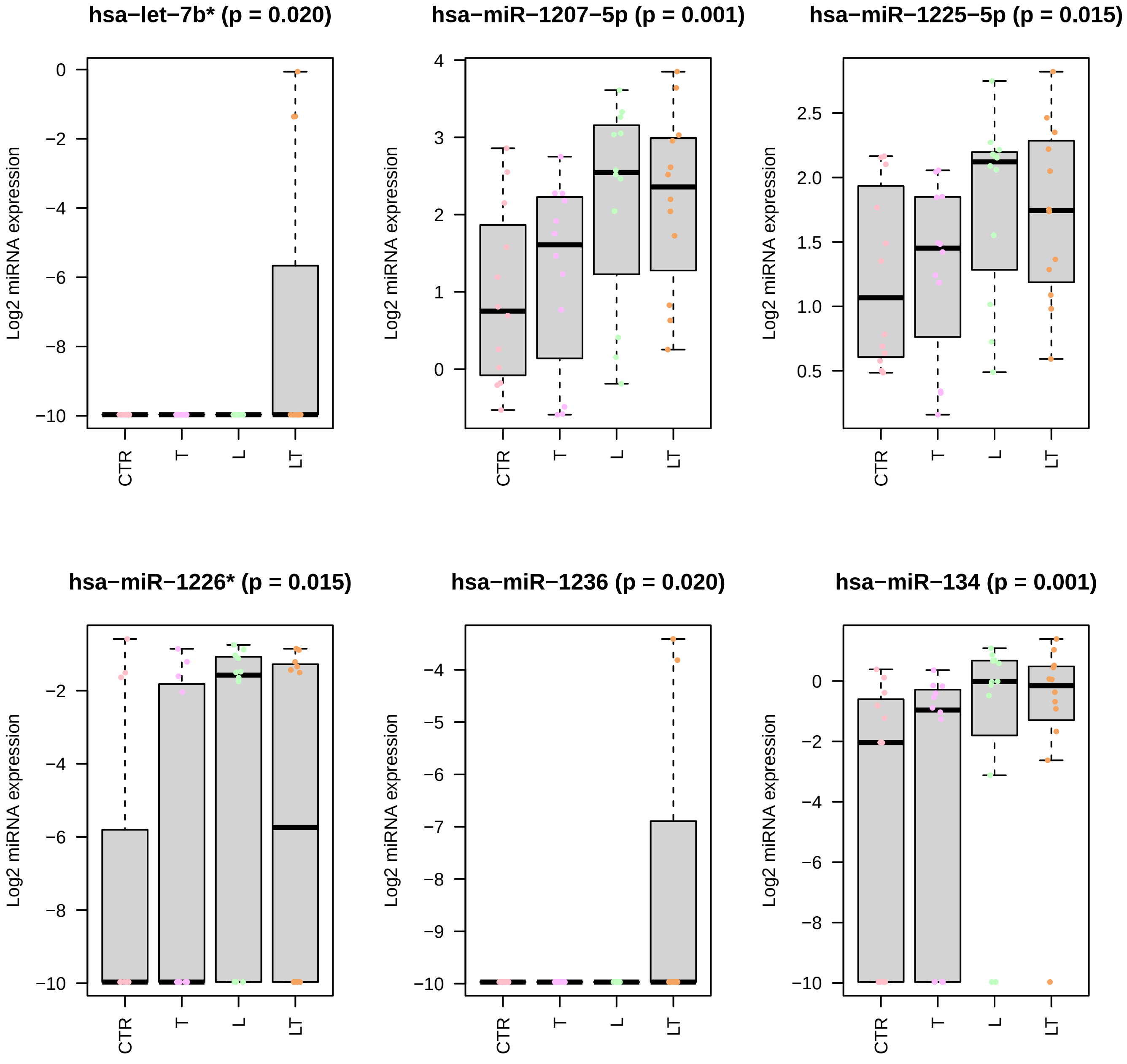

Supplementary Figure S2: miRNA expression before and after targeted treatment in responsive cell lines (SKBR3, BT-474)

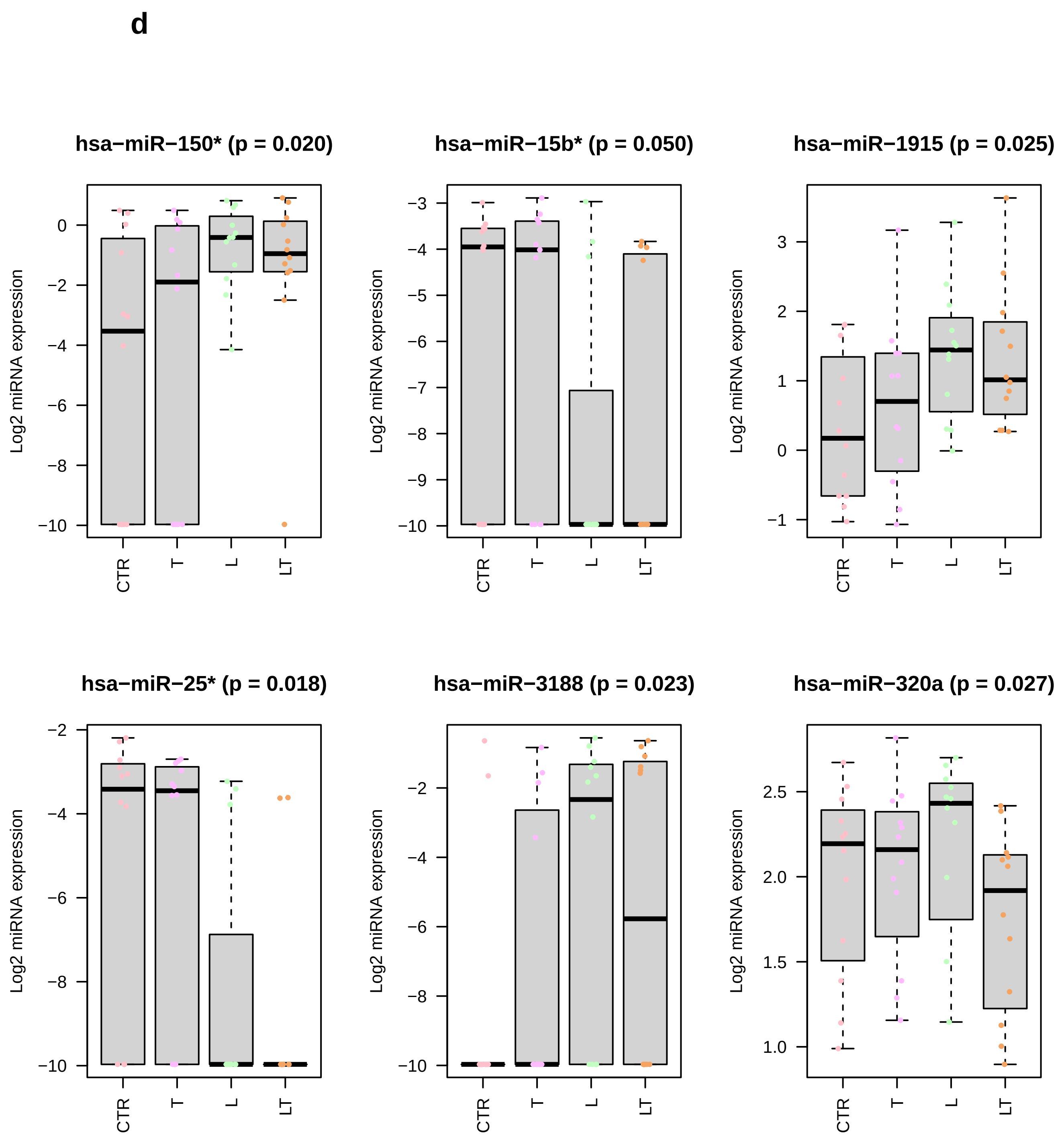

Supplementary Figure S2: miRNA expression before and after targeted treatment in responsive cell lines (SKBR3, BT-474)

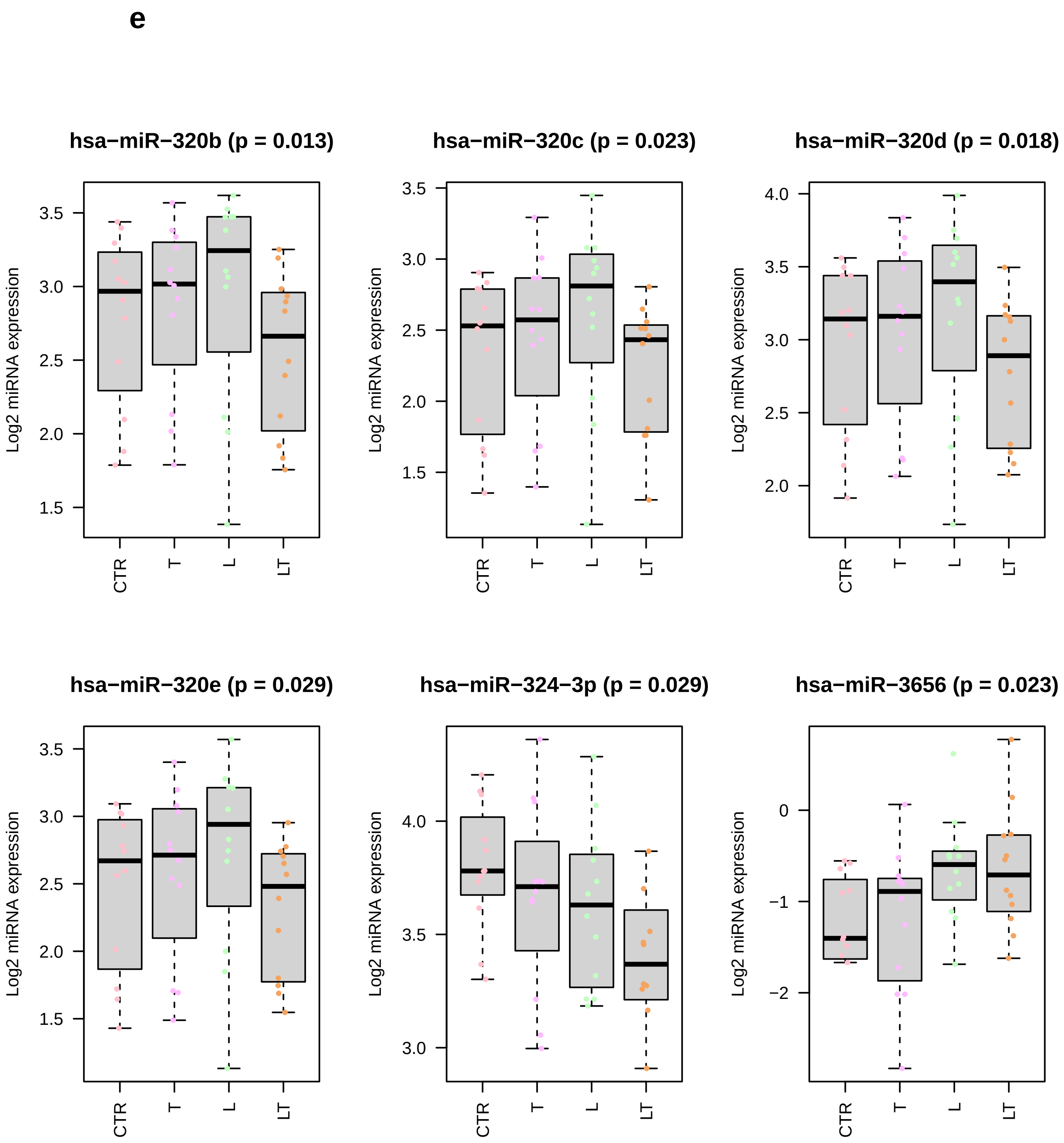

Supplementary Figure S2: miRNA expression before and after targeted treatment in responsive cell lines (SKBR3, BT-474)

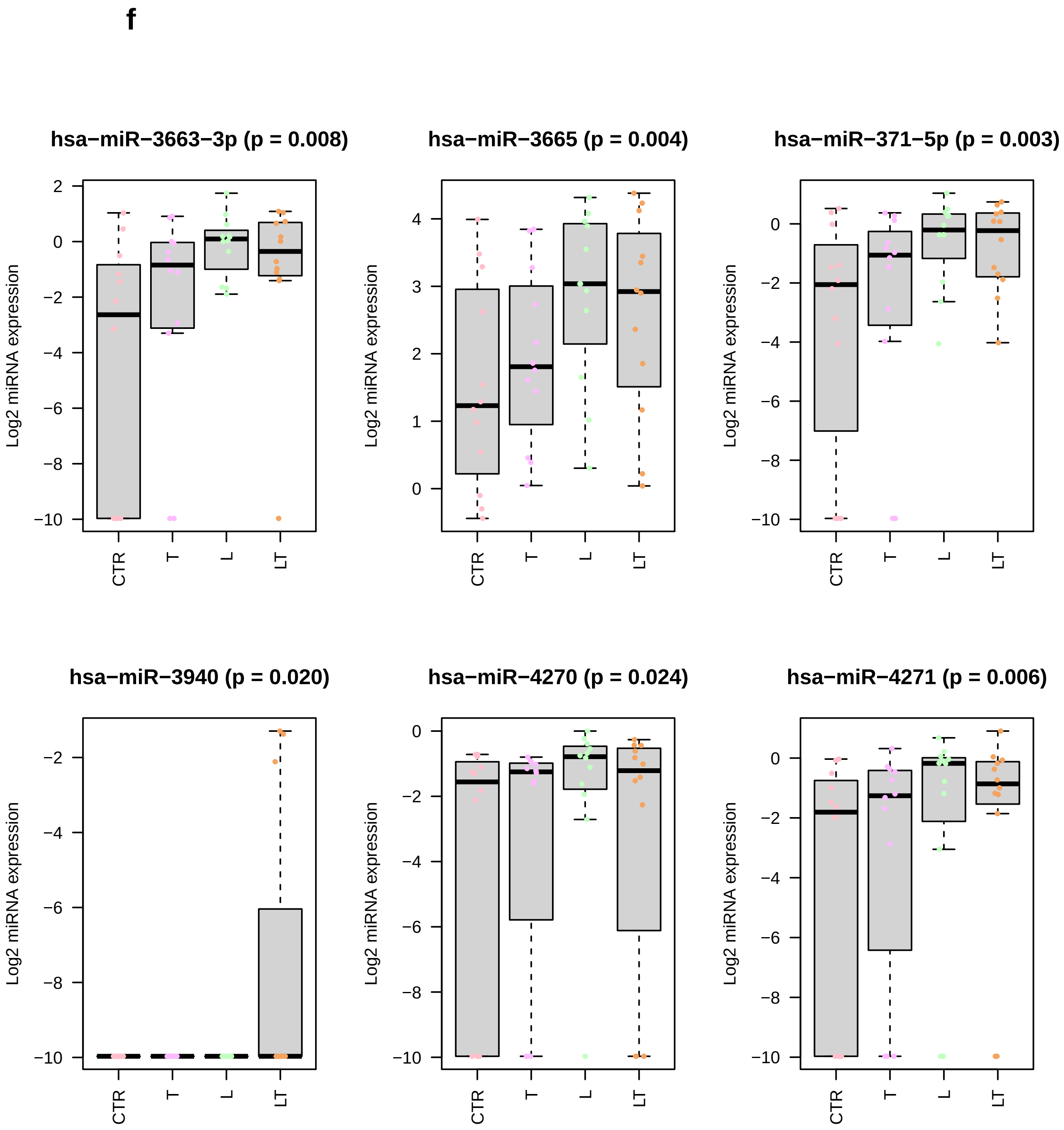

Supplementary Figure S2: miRNA expression before and after targeted treatment in responsive cell lines (SKBR3, BT-474)

**g**

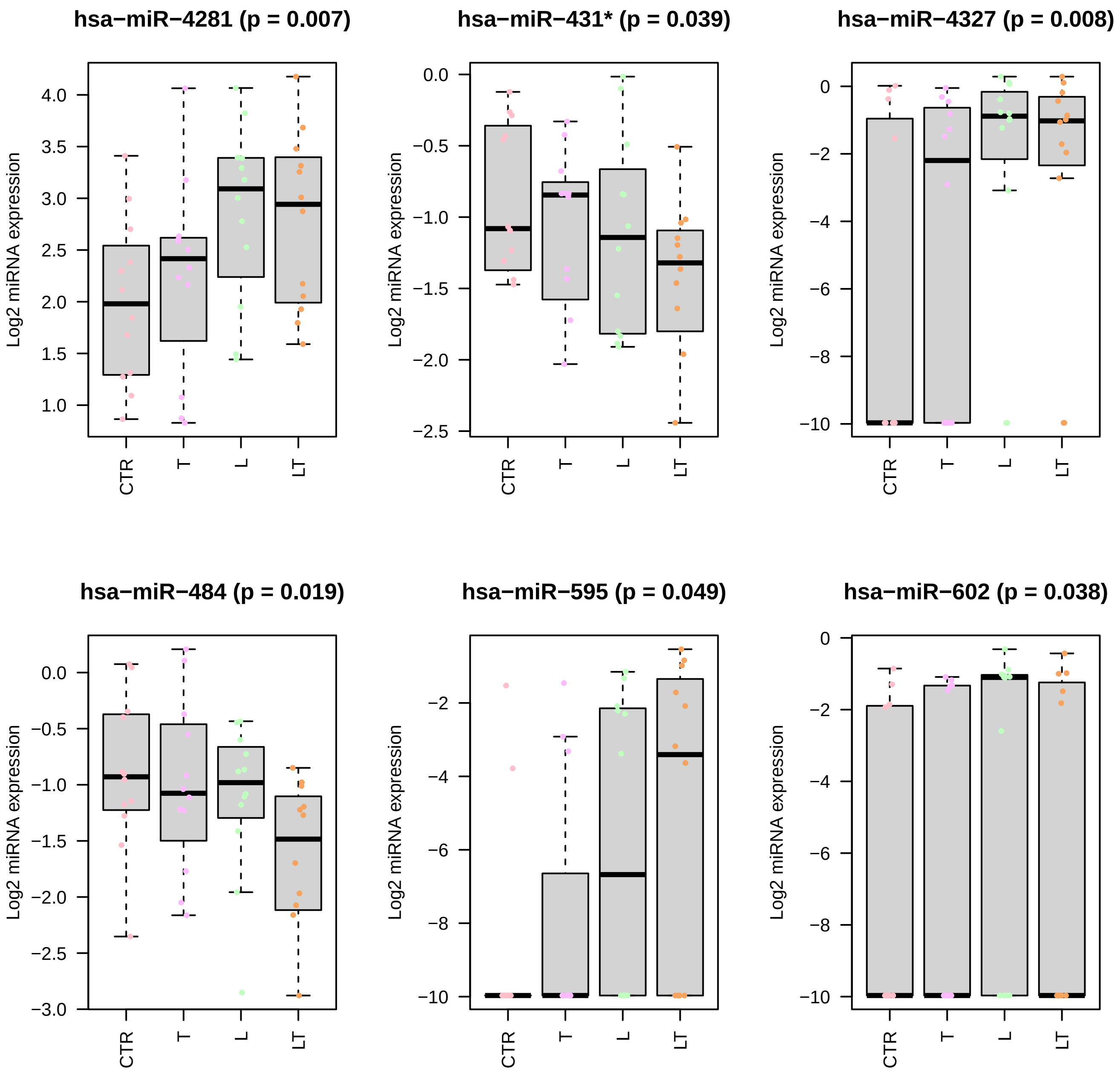

Supplementary Figure S2: miRNA expression before and after targeted treatment in responsive cell lines (SKBR3, BT-474)

**h**

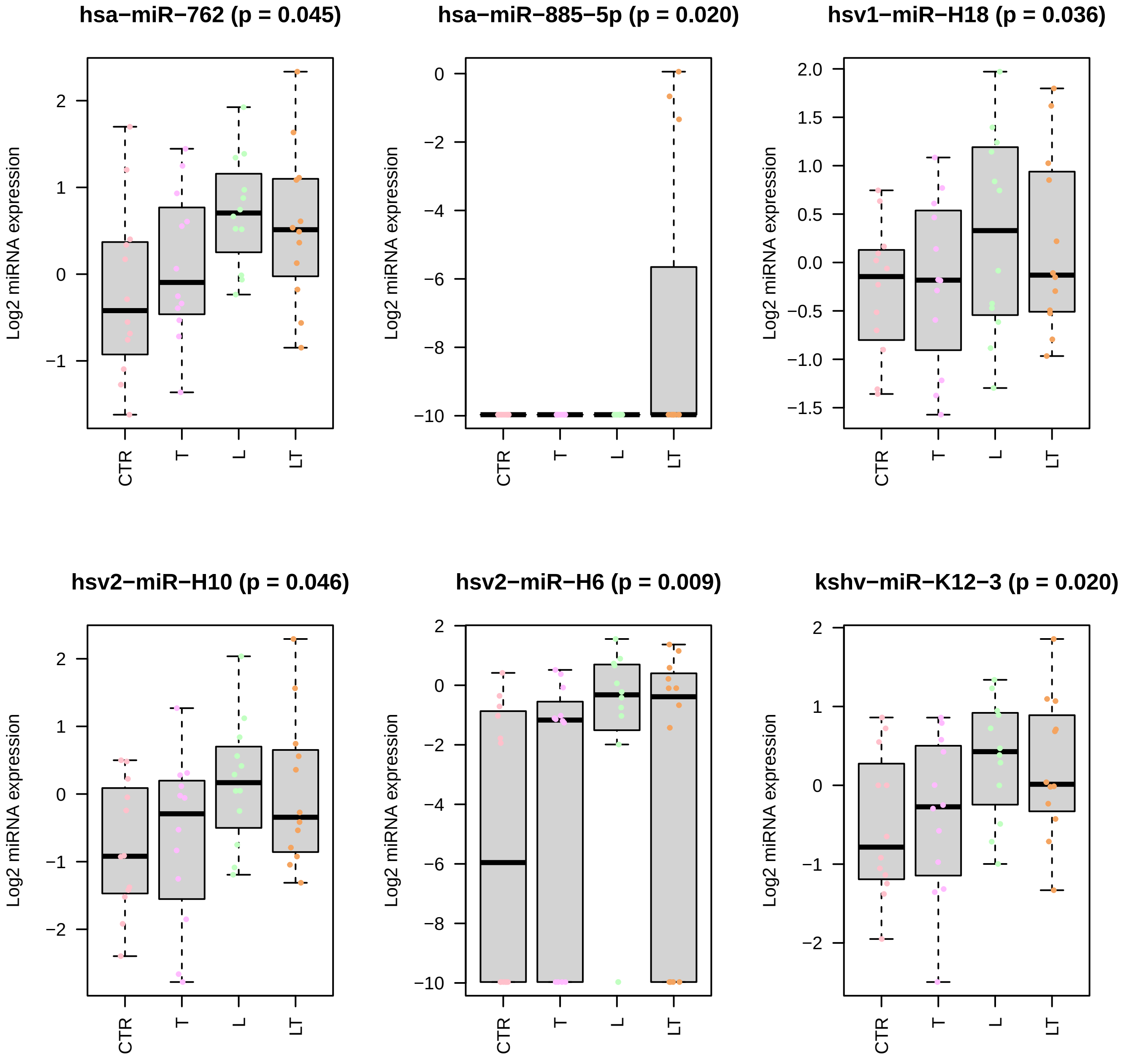

Supplementary Figure S2: miRNA expression before and after targeted treatment in poorly responsive cell lines (KPL4. SUM190PT)

**i**

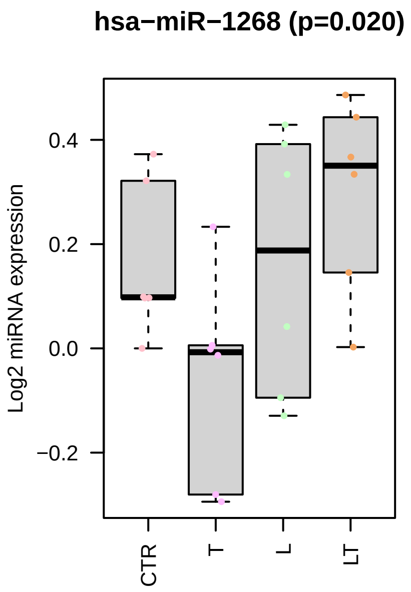

# Supplementary Figure S3

**a**

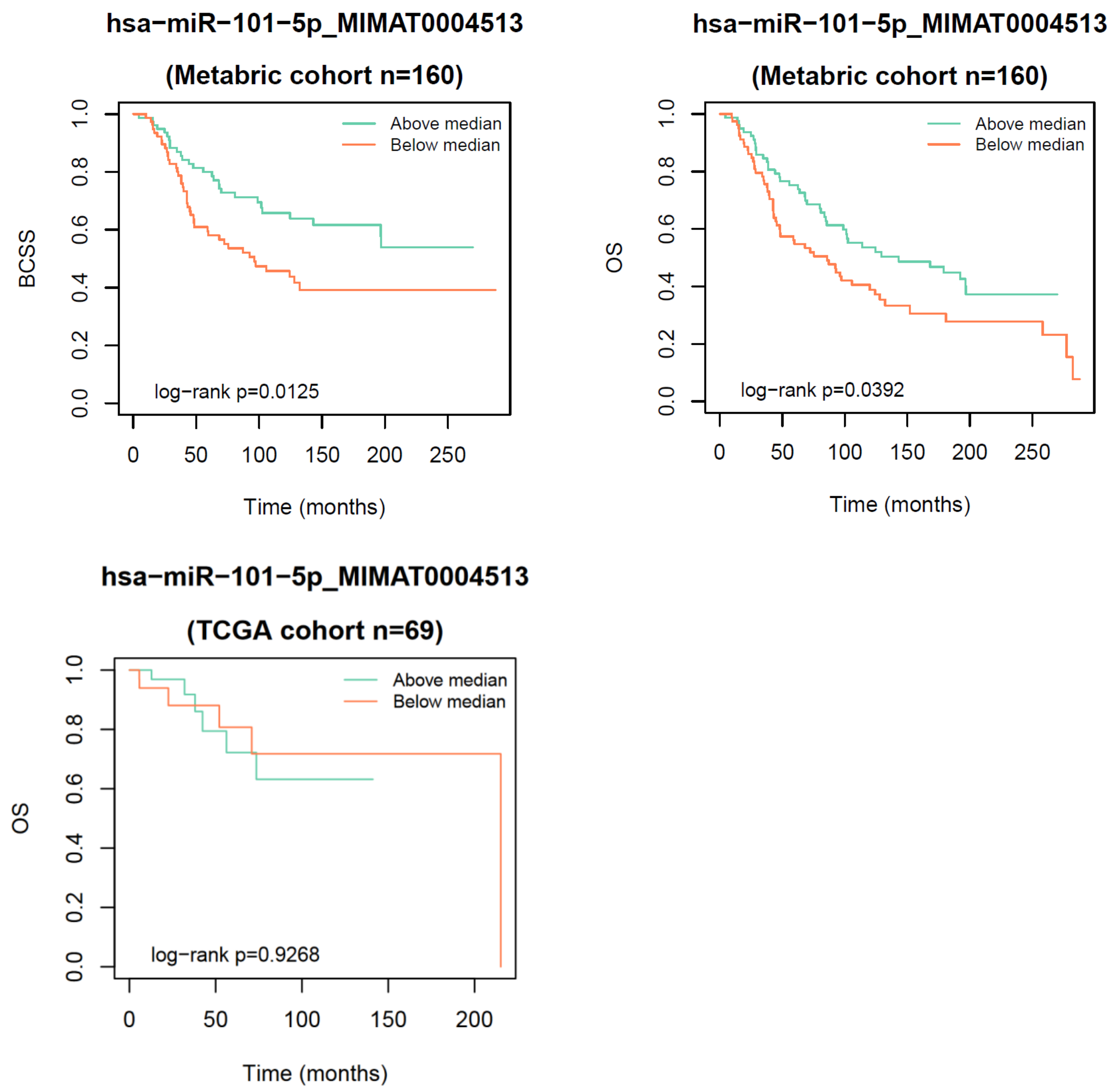


**Supplementary Figure S3: The clinical significance of sensitizing miRNAs in breast cancer patients**.

The survival curves are prepared for HER2+ breast cancer patients in the METABRIC ($n$ = 160) and the TCGA($n$ = 69) datasets. For the METABRIC data, breast cancer specific survival (BCSS) and overall survival (OS) of four miRNAs are presented (miR-101-5p (a), miR-29a-3p (b), miR29c-3p (c) and miR-744-3p (d)). For the TCGA data, OS for five miRNAs are presented (miR-101-5p (a), miR-29a-3p (b), miR29c-3p (c), miR-744-3p (d) and mir-106a-5p (e)). Log-rank $p$-values are significant ($p < 0.05$) only for miR-101-5p in the METABRIC dataset.

Supplementary Figure S3

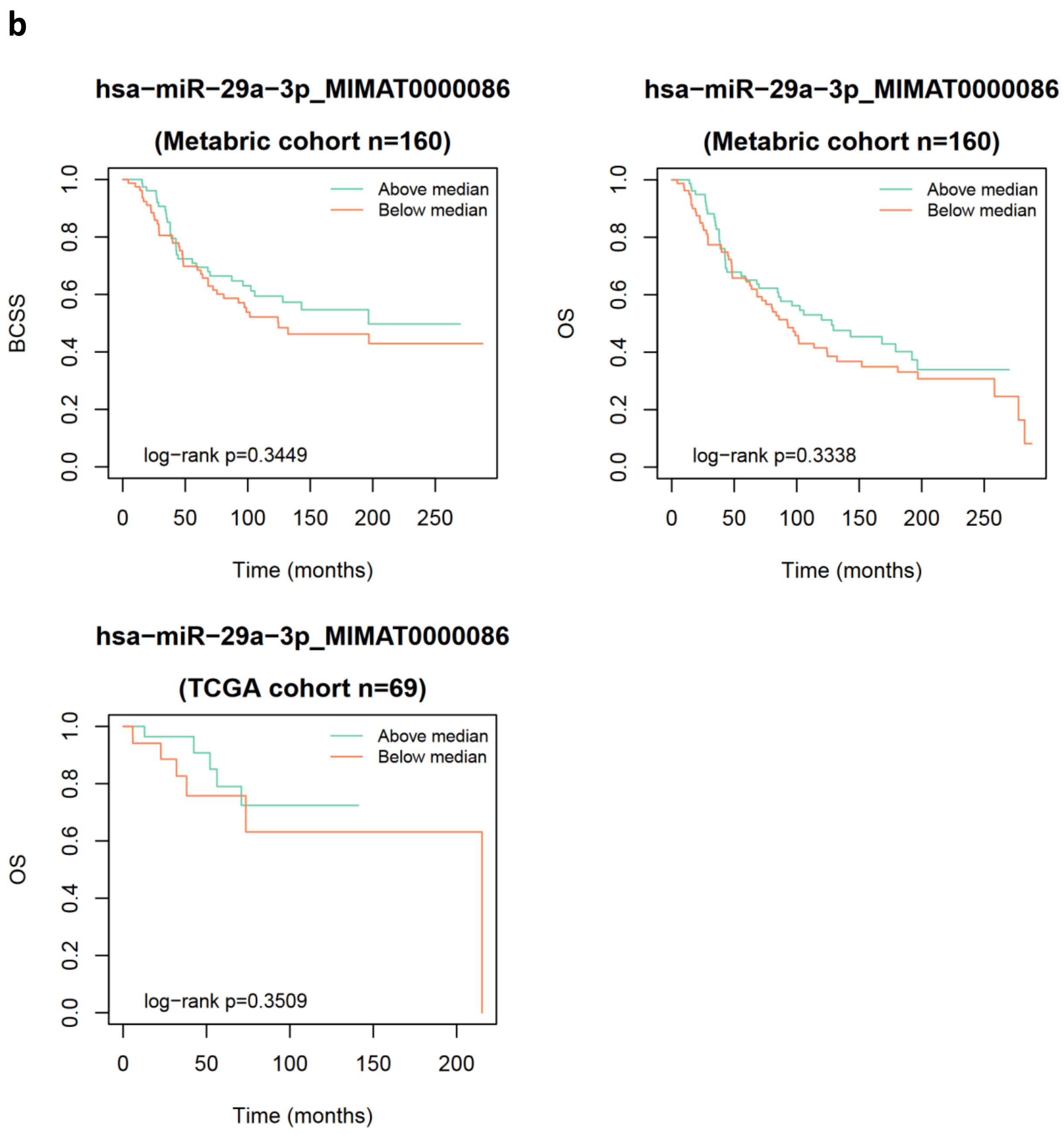

# Supplementary Figure S3

**c**

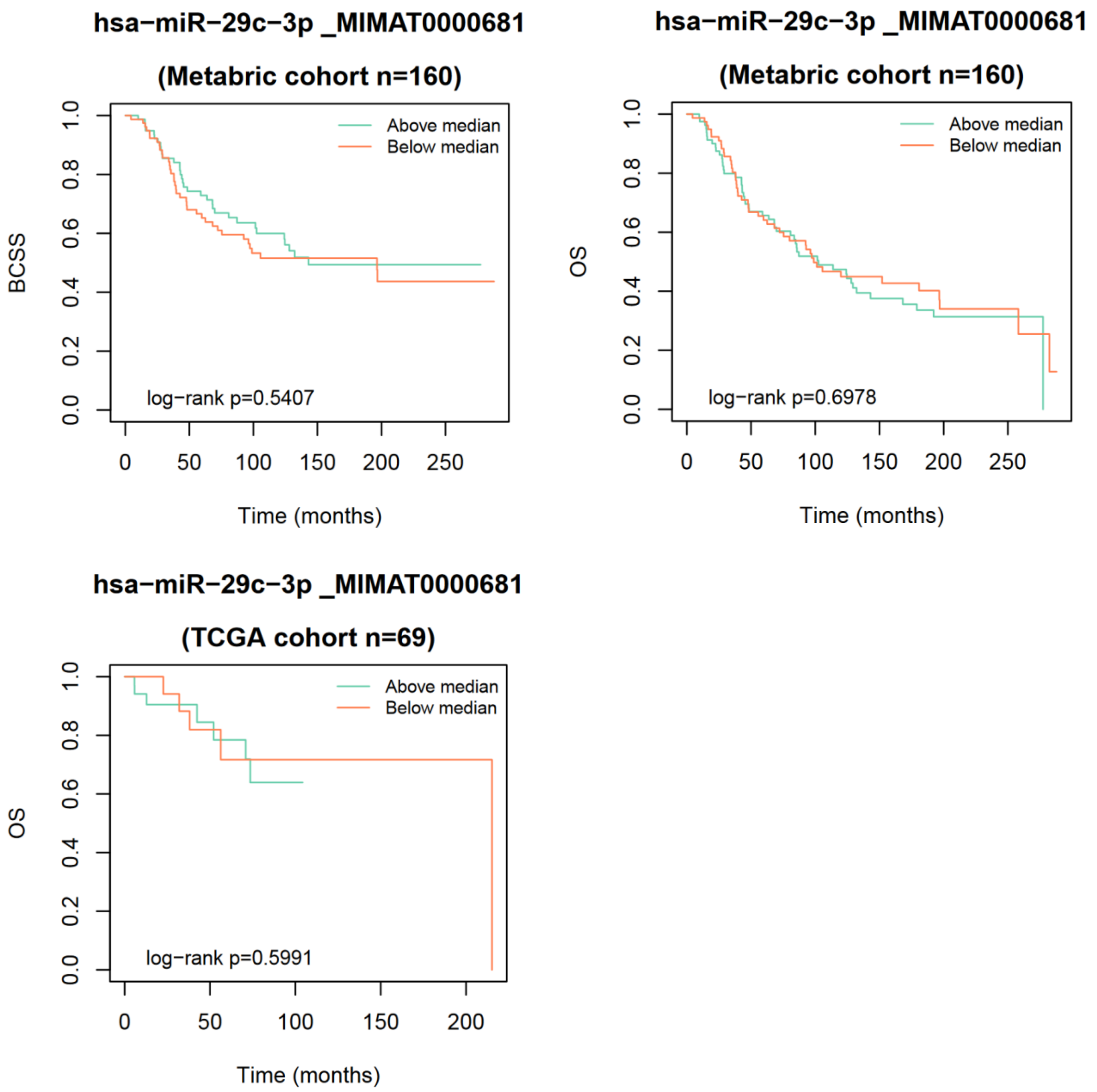

Supplementary Figure S3

**d**

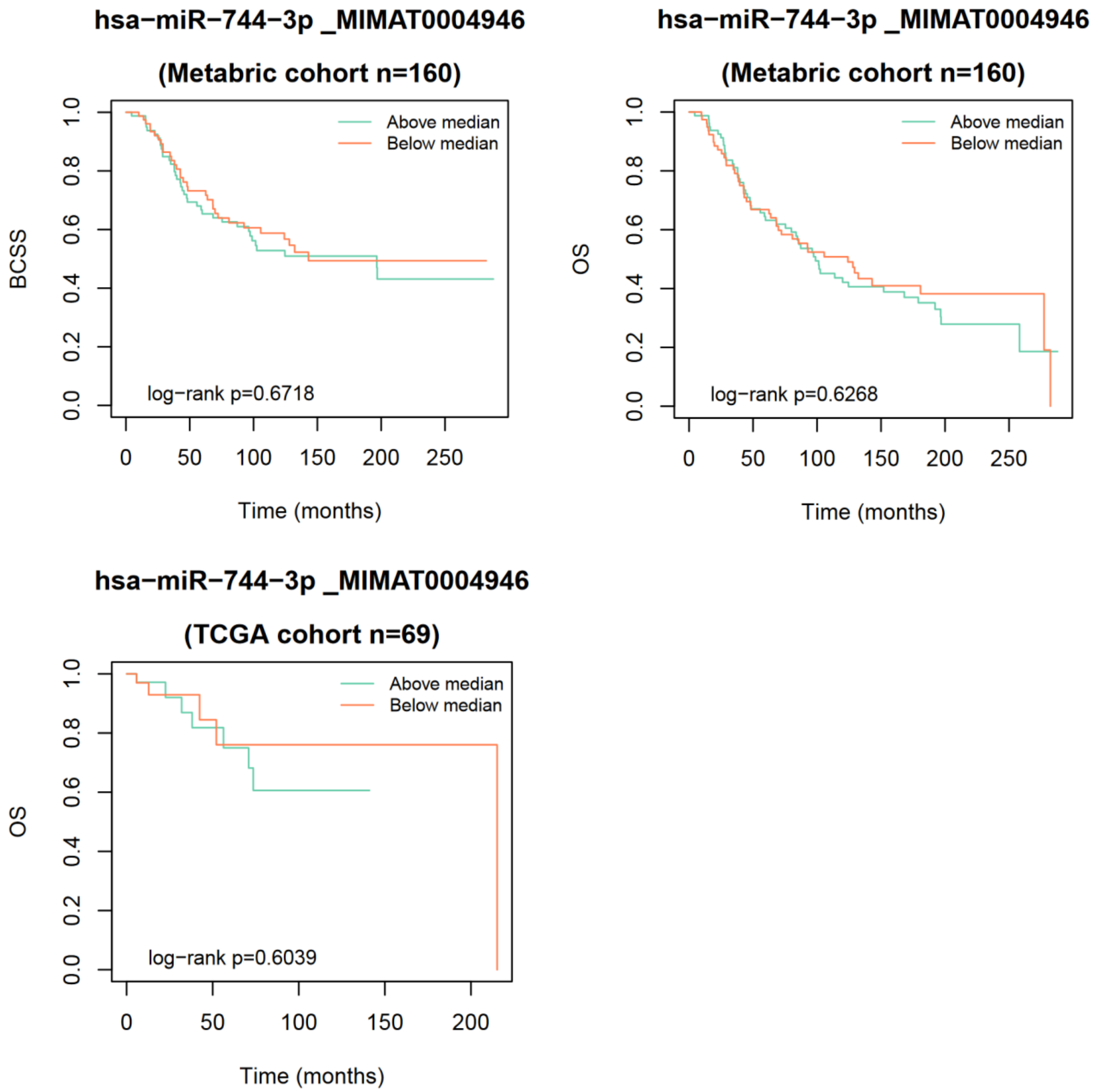

Supplementary Figure S3

**e**

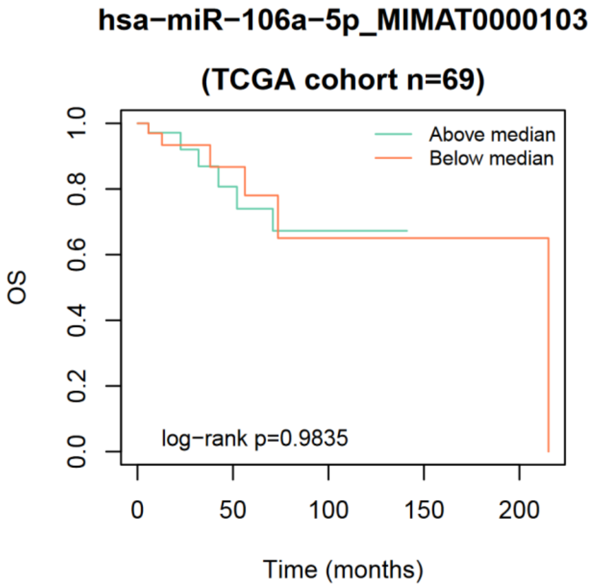

Supplement: Supplementary file 2 — Supplementary Information 2. [file 41598_2021_90385_MOESM2_ESM.pdf]
